# Effects of soluble CPE on glioma cell migration are associated with mTOR activation and enhanced glucose flux

**DOI:** 10.18632/oncotarget.18747

**Published:** 2017-06-27

**Authors:** Elena I. Ilina, Angela Armento, Leticia Garea Sanchez, Marina Reichlmeir, Yannick Braun, Cornelia Penski, David Capper, Felix Sahm, Lukas Jennewein, Patrick N. Harter, Sven Zukunft, Ingrid Fleming, Dorothea Schulte, Francois Le Guerroué, Christian Behrends, Michael W. Ronellenfitsch, Ulrike Naumann, Michel Mittelbronn

**Affiliations:** ^1^ Institute of Neurology (Edinger Institute), Goethe University, 60528 Frankfurt, Germany; ^2^ Luxembourg Centre of Neuropathology (LCNP), 3555 Dudelange, Luxembourg; ^3^ NORLUX Neuro-Oncology Laboratory, Department of Oncology, Luxembourg Institute of Health (L.I.H.), 1526 Luxembourg, Luxembourg; ^4^ Molecular Neurooncology, Department of Vascular Neurology, Hertie Institute for Clinical Brain Research and Center Neurology, University of Tübingen, 72076 Tübingen, Germany; ^5^ German Cancer Consortium (DKTK), German Cancer Research Center (DKFZ), 69120 Heidelberg, Germany; ^6^ Department of Neuropathology, Institute of Pathology, Ruprecht-Karls-University, 69120 Heidelberg, Germany; ^7^ Institute for Vascular Signaling, Centre for Molecular Medicine, Goethe University, 60590 Frankfurt, Germany; ^8^ Institute of Biochemistry II, Medical School Goethe University, 60528 Frankfurt, Germany; ^9^ Munich Cluster for Systems Neurology (SyNergy), Medical Faculty, Ludwig-Maximilians-University (LMU) Munich, 81377 Munich, Germany; ^10^ Senckenberg Institute of Neurooncology, Goethe University, 60528 Frankfurt, Germany; ^11^ Laboratoire National de Santé, Department of Pathology, 3555 Dudelange, Luxembourg; ^12^ Luxembourg Centre for Systems Biomedicine (LCSB), University of Luxembourg, 4361 Esch-sur-Alzette, Luxembourg

**Keywords:** CPE, glioblastoma, migration, metabolism, mTOR

## Abstract

Carboxypeptidase E (CPE) has recently been described as a multifunctional protein that regulates proliferation, migration and survival in several tumor entities. In glioblastoma (GBM), the most malignant primary brain tumor, secreted CPE (sCPE) was shown to modulate tumor cell migration. In our current study, we aimed at clarifying the underlying molecular mechanisms regulating anti-migratory as well as novel metabolic effects of sCPE in GBM. Here we show that sCPE activates mTORC1 signaling in glioma cells detectable by phosphorylation of its downstream target RPS6. Additionally, sCPE diminishes glioma cell migration associated with a negative regulation of Rac1 signaling via RPS6, since both inhibition of mTOR and stimulation of Rac1 results in a reversed effect of sCPE on migration. Knockdown of CPE leads to a decrease of active RPS6 associated with increased GBM cell motility. Apart from this, we show that sCPE enhances glucose flux into the tricarboxylic acid cycle at the expense of lactate production, thereby decreasing aerobic glycolysis, which might as well contribute to a less invasive behavior of tumor cells. Our data contributes to a better understanding of the complexity of GBM cell migration and sheds new light on how tumor cell invasion and metabolic plasticity are interconnected.

## INTRODUCTION

Glioblastoma (GBM) is the most common and aggressive tumor of glial origin in the adult brain. One of the main hallmarks of GBM, diffuse infiltration of glioma cells into the brain parenchyma, renders complete neurosurgical resection of the tumor impossible. Furthermore, a fast metabolic adaptation to the rapidly changing GBM microenvironment may be at least partly responsible for treatment resistance and, consequently, tumor recurrence [1–3]. Therefore, despite maximum treatment including neurosurgical resection combined with adjuvant chemo- and radiotherapy, median survival of patients nowadays still does not exceed 15 months even in patients in good clinical condition [4, 5]. At the microscopic level, glioblastoma consists of highly proliferative foci as well as diffusely infiltrating cells at the tumor border zones. These two processes are considered mutually exclusive at single cell level, which has led to the so-called “go or grow” concept [6, 7]. Within recent years, several factors involved in the differential regulation of the “go or grow” processes, have been identified, including distinct miRNAs [8], reciprocal transcription factor activation [9] or the metabolic switch between glycolysis and pentose-phosphate pathway [10, 11], highlighting multiple facets of this concept. Recently, we showed that carboxypeptidase E (CPE) can modulate the dichotomic process of migration and proliferation in GBM cells [12]. Originally described as an enkephalin convertase [13–15], CPE was later shown to be involved in structural organization of the cell (e.g. as a sorting receptor [16], vesicle anchorage [17] and organization of synaptic vesicles [18] as well as in survival of certain neuronal populations [19, 20]). Additionally, soluble CPE (sCPE) has been implicated in regulation of proliferation, migration and survival of cancer cells of pheochromocytoma, fibrosarcoma, hepatocellular carcinoma [21] and among others glioma [12]. Although some functional effects have already been deciphered for sCPE, only little mechanistic data exists so far, especially for the anti-migratory effects of sCPE. Considering the involvement of sCPE in sorting and maturation of metabolically active neuropeptides and a growing body of interest regarding the association of glioma cell migration and/or invasion with certain metabolic adaptation processes [1], we aimed at deciphering the underlying mechanistic role of CPE in the regulation of cell motility and metabolic plasticity in glioma. Therefore, we generated stable sCPE-overexpressing glioma cell lines as well as GBM CPE knockdown cells and subsequently performed phospho-proteomic analysis to study potential downstream targets. We further utilized a panel of seven GBM cell lines that differed in secreted CPE levels to confirm selected downstream targets. We show that sCPE leads to phosphorylation of the mTORC1 target ribosomal protein S6 (RPS6) and we propose, that this activation of RPS6 negatively regulates Rac1-signaling thereby attenuating the migratory behavior of GBM cells. Both inhibition of mTOR and activation of Rac1 result in reduced anti-migratory effects of sCPE. Additionally, Rac1 activation attenuates phosphorylation of mTORC1 target RPS6, suggesting a negative feedback to the mTOR pathway. Moreover, sCPE induces a distinct metabolic phenotype in glioma cells by increasing the tricarboxylic acid cycle (TCA) flux but diminishing aerobic glycolysis and, consequently, lactate production, a known pro-migratory metabolite. Hence, we propose, that sCPE enhances RPS6 activation via mTOR signaling and reduces aerobic glycolysis thereby constituting a novel modulator of glioma cell migration.

## RESULTS

### sCPE activates the central mTORC1 target RPS6

**Figure 1:**
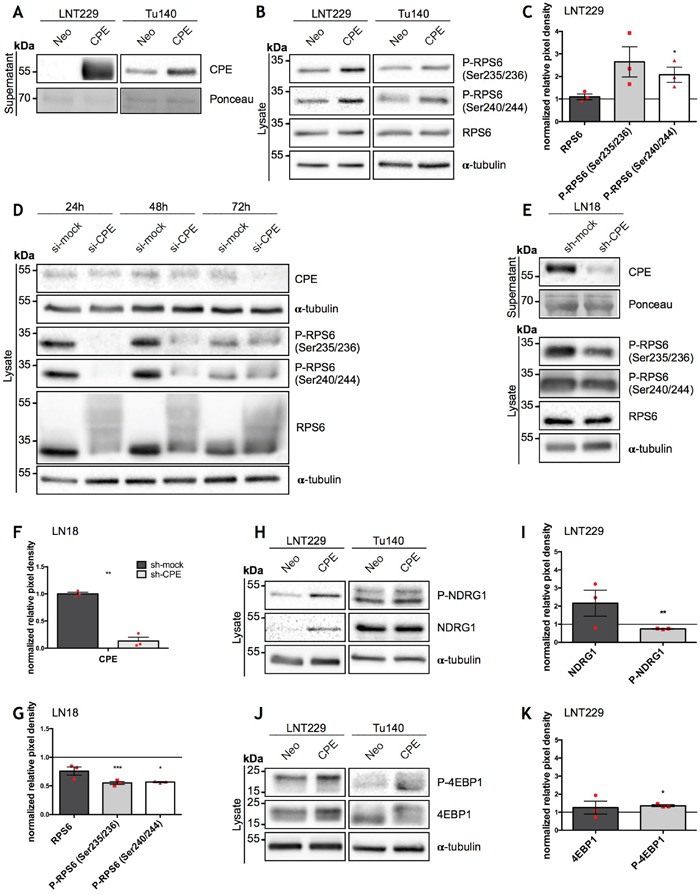
Overexpression of sCPE results in mTORC1 activation while CPE knockdown leads to decrease of its activity **(A)** Immunoblot of sCPE obtained from the supernatants of the sCPE-transfected LNT229 and Tu140 cells. Supernatants derived from Neo LNT229 cells were used as control. Ponceau staining was used as a loading control. The cells were serum-starved for 24h in serum-reduced medium prior to supernatant collection. A representative immunoblot is shown. **(B, H, J)** Representative immunoblots for detection of phosphorylated and total amounts of RPS6 **(B)**, NDRG1 **(H)** and 4EBP1 **(J)** in the sCPE-overexpressing vs. Neo LNT229 and Tu140 cell lysates. α-tubulin was used as a loading control. The cells were serum-starved for 24h in serum-reduced medium prior to lysis. **(C, I, K)** Quantification of densitometric measurements of immunoblotting results of total as well as phosphorylated amounts of **(C)** RPS6, **(I)** NDRG1 and **(K)** 4EBP1 in the sCPE-overexpressing vs. Neo LNT229 cells (normalized to Neo LNT229 cells; set to 1). Red dots represent single experiments. Ratio-based paired t-test. Mean±SEM; n=3 (C: *p=0.0482; F: **p=0.0052; H: *p=0.0148). **(D)** A representative immunoblot for detection of CPE as well as phosphorylated and total amounts of RPS6 in the lysates of the transient CPE-knockdown primary GBM Tu140 cells. Control siRNA (si-mock) was used as negative control. α-tubulin was used as a loading control. **(E)** A representative immunoblot for detection of secreted CPE in the supernatants as well as phosphorylated and total amounts of RPS6 in the lysates of the stable CPE-knockdown LN18 cells. Control shRNA (sh-mock) was used as negative control. Prior to supernatant collection, the cells were serum-starved for 24h in serum-reduced medium. Ponceau and α-tubulin were used as loading controls in the supernatant- and in the lysate, respectively. **(F, G)** Quantification of densitometric measurements of immunoblotting results of CPE- **(F)** as well as total and phosphorylated amounts of RPS6 **(G)** protein levels in the LN18 cell line upon CPE knockdown (in G: RPS6 levels in LN18 sh-CPE cells normalized to LN18 sh-mock cells). Red dots represent single experiments. Ratio-based paired t-test. Mean±SEM; n=3 (F: **p=0.0021; G: ***p=0.0006, *p=0.0439).

Since the underlying mechanisms of how sCPE regulates glioma cell migration are still unclear and cellular regulatory pathways frequently transmit signals through phosphorylation cascades, we first explored the phospho-proteome of sCPE-overexpressing- versus corresponding control LNT229 GBM cells (Neo) in order to detect possible targets of sCPE. We identified several targets of the mTOR pathway with increased phosphorylation in sCPE-overexpressing- compared to LNT229 Neo cells (Table 1; the unprocessed list of detected proteins is available in Supplementary Table 1 or on *MassIVE^#^*). According to the mass-spectrometry data, sCPE activated signaling from both mTOR complexes (mTORC1 and mTORC2) as evidenced by an increase in phosphorylation of the indirect targets RPS6 and N-Myc Downstream Regulated 1 (NDRG1), respectively [22, 23]. In addition, the regulator of cytoskeleton stability Cofilin-1 was also hyperphosphorylated at Ser3 (Table 1), indicating its inactivated state and therefore, potentially, a lesser contribution to the migratory phenotype of the glioma cells [24]. By contrast, known pro-invasive proteins, for instance, kinesin light chain, showed weaker phosphorylation (Table 2) [25]. To confirm the phospho-proteomic data, we performed western blot analysis of CPE-overexpressing- versus Neo LNT229 cells (Figure 1A). Indeed, we found a stronger phosphorylation of RPS6 protein when sCPE was overexpressed (Figure 1B), especially with regard to the Ser240/244 phosphorylation motif (Figure 1C). This effect was further confirmed using primary Tu140 GBM cells, in which we also overexpressed sCPE (Figure 1A, 1B). Of note, due to the primary origin of the Tu140 cells, overexpression of sCPE was only stable for 1 passage (corresponds to the supernatants and respective lysates on the Figure 1A, 1B) and lost on subsequent passages, making quantification impossible. Remarkably, siRNA-mediated transient knockdown of CPE in wild-type Tu140 cells, which normally secrete large amounts of CPE, revealed a decrease in active RPS6 via reduction in both phosphorylation and total protein (Figure 1D, Supplementary Figure 1A, 1B). shRNA-mediated stable CPE knockdown in LN18 GBM cell line revealed reduction in the active RPS6 as well, however, not affecting the total amount of protein (Figure 1E, 1F, 1G, Supplementary Figure 1C). The total amount of NDRG1 as well as its phosphorylation was induced, however quantification of phospho-to-total ratio of NDRG1 protein revealed a significant reduction in phosphorylation in LNT229 cells upon sCPE overexpression (Figure 1H, 1I). Meanwhile, no considerable changes in phosphorylated NDRG1 have been observed in the Tu140 sCPE-overexpressing cells. Moreover, knockdown of CPE in Tu140 cells resulted in even higher phosphorylation of NDRG1 (Supplementary Figure 2), altogether not supporting the mass-spectrometry data. Apart from RPS6, mTORC1 phosphorylates 4EBP1. We further investigated if overexpression of sCPE also promoted phosphorylation of 4EBP1. Indeed, total amount of 4EBP1 as well as its phosphorylation were elevated in both LNT229 and Tu140 cells (Figure 1J, 1K). However, knockdown of CPE in Tu140 GBM cells showed no gross effect on 4EBP1 (Supplementary Figure 2), suggesting that sCPE mainly activates RPS6 only.

**Table 1:** Hyperphosphorylated proteins in LNT229 glioma cells upon sCPE overexpression

H/L	A.a.	Position	Loc.Prob.	Protein Name
**1.90**	**T**	**37;75;83;156;258;247;262;328**	**0.91**	**Protein NDRG1**
**1.88**	**S**	**204;235**	**1.00**	**40S ribosomal protein S6 (RPS6)**
1.82	S	569	1.00	Nucleolar protein 56 (NOP56)
1.82	S	570	1.00	Nucleolar protein 56 (NOP56)
**1.81**	**S**	**42;80;88;161;263;252;267;333**	**0.98**	**Protein NDRG1**
**1.74**	**S**	**45;83;91;164;266;255;270;336**	**0.71**	**Protein NDRG1**
1.74	T	773;822;860;939;977;904;909	0.57	Transcriptional regulator ATRX
1.70	S	3;3	1.00	Destrin (DSTN)
**1.68**	**T**	**44;82;90;163;265;254;269;335**	**0.77**	**Protein NDRG1**
1.57	S	171;219;296;314;271;307;336; 338;365;367;396;713;731	0.82	Microtubule-associated protein tau (MAPT)
1.57	S	179;227;304;322;279;315;344; 346;373;375;404;721;739	0.95	Microtubule-associated protein tau (MAPT)
1.57	T	178;226;303;321;278;314;343; 345;372;374;403;720;738	0.91	Microtubule-associated protein tau (MAPT)
**1.50**	**S**	**3**	**1.00**	**Cofilin-1 (CFL1)**
1.25	S	12;264;290	0.86	Nuclear factor 1; Nuclear factor 1 B-type (NFIB)
1.22	T	458	0.90	Vimentin (VIM)
1.22	S	99	1.00	Lamin-B receptor (LBR)
1.21	S	214;691;888;922;1095;877;915;933;991;996;1099	0.69	Pleckstrin homology domain-containing family A member 5 (PLEKHA5)
1.21	S	220;697;894;928;1101;883;921;939;997;1002;1105	0.87	Pleckstrin homology domain-containing family A member 5 (PLEKHA5)
1.21	S	615;664;702;781;819;746;751	0.96	Transcriptional regulator ATRX
1.17	S	270	1.00	Thioredoxin-related transmembrane protein 1 (TMX1)
1.17	S	39;77;85;158;260;249;264;330	0.99	Protein NDRG1
1.13	S	231;240;348;246;323;335	0.70	Protein NDRG3
1.09	S	220	0.88	Neuroblast differentiation-associated protein AHNAK
1.09	T	234;205	1.00	Nucleophosmin NPM1
1.09	T	237;208	0.99	Nucleophosmin NPM1
1.08	S	1066;1068	1.00	general transcription factor IIIC subunit 1 (GTF3C1)
1.08	S	12	0.70	Sulfate transporter SLC26A2
1.08	S	16	0.54	Sulfate transporter SLC26A2
1.08	S	152;134;172;190	0.86	Melanoma-associated antigen D2 (MAGED2)
1.08	S	156;138;176;194	1.00	Melanoma-associated antigen D2 (MAGED2)

The table provides a list of hyperphosphorylated proteins in CPE-overexpressing LNT229 versus corresponding Neo-control cells detected by SILAC followed by phosphoproteomic analysis. The phospho-proteins discussed and studied in the manuscript are highlighted in bold. Abbreviations: H/L - log2 ratio of phospho-enrichment in “heavy”-labeled CPE-overexpressing LNT229 cells to “light”-labeled Neo-control LNT229 cells; A.a. - amino acid; Position - position of amino acid with detected phosphorylation; Loc.Prob. - localization probability.

**Table 2:** Hypophosphorylated proteins in LNT229 glioma cells upon sCPE overexpression

H/L	A.a.	Position	Loc.Prob.	Protein Name
−3.39	S	4	1.00	Heterogeneous nuclear ribonucleoprotein A1 (HNRNPA1)
−2.62	S	66;73;78;71;60;225;65	0.60	LIM and calponin homology domains-containing protein 1 (LIMCH1)
−2.62	S	67;74;79;72;61;226;66	0.60	LIM and calponin homology domains-containing protein 1 (LIMCH1)
−2.62	S	72;79;84;77;66;231;71	0.62	LIM and calponin homology domains-containing protein 1 (LIMCH1)
−2.51	S	224;126	0.99	Trans-Golgi network integral membrane protein 2 (TGOLN2)
−2.15	S	390;439;477;556;594	0.93	Transcriptional regulator ATRX
−2.15	S	392;441;479;558;596	0.55	Transcriptional regulator ATRX
−1.98	S	74;81;86;79;68;233;73	0.70	LIM and calponin homology domains-containing protein 1 (LIMCH1)
−1.95	S	123	1.00	RING1 and YY1-binding protein (RYBP)
−1.91	S	366	1.00	Protein CASC4
−1.91	S	374	1.00	Protein CASC4
−1.91	S	696;266	0.88	SWI/SNF-related matrix-associated actin-dependent regulator of chromatinsubfamily A containing DEAD/H box 1 (SMARCAD1)
−1.91	Y	703;273	0.97	SWI/SNF-related matrix-associated actin-dependent regulator of chromatinsubfamily A containing DEAD/H box 1 (SMARCAD1)
−1.51	S	378	1.00	Integrator complex subunit 12 (INTS12)
−1.50	S	181;191	1.00	Golgi membrane protein 1 (GOLM1)
−1.30	S	167;166;69	1.00	ATP-binding cassette sub-family F member 1 (ABCF1)
−1.29	S	55	0.85	Signal-induced proliferation-associated protein 1 (SIPA1)
−1.28	S	317	1.00	Cyclin-dependent kinase 13 (CDK13)
−1.28	S	325	0.97	Cyclin-dependent kinase 13 (CDK13)
−1.28	S	1785	0.99	Microtubule-associated protein 1B (MAP1B)
−1.26	S	39;134	0.62	LEM domain-containing protein 2 (LEMD2)
−1.26	S	44;139	0.85	LEM domain-containing protein 2 (LEMD2)
−1.24	S	50;357;480;485	1.00	Apoptosis-stimulating of p53 protein 2 (TP53BP2)
**-1.23**	**S**	**512;589**	**1.00**	**Kinesin light chain 2 (KLC2)**
−1.22	S	1779	0.97	Microtubule-associated protein 1B (MAP1B)
−1.20	S	180	0.57	Insulin-like growth factor 2 mRNA-binding protein 3 (IGF2BP3)
−1.19	S	152	0.84	YTH domain-containing protein 1 (YTHDC1)
−1.18	S	58;65;70;63;52;217;57	1.00	LIM and calponin homology domains-containing protein 1 (LIMCH1)
−1.18	T	63;68;61;50;215;55;56	0.99	LIM and calponin homology domains-containing protein 1 (LIMCH1)
−1.18	S	24;148;449;456;486;488;508;525;542	0.76	Histone deacetylase 7 (HDAC7)
−1.16	S	102	0.73	PC4 and SFRS1-interacting protein (PSIP1)
−1.16	S	105	0.67	PC4 and SFRS1-interacting protein (PSIP1)
−1.16	S	196;202;207	1.00	Caldesmon (CALD1)
−1.16	S	977	1.00	Protein-methionine sulfoxide oxidase MICAL3
−1.15	S	1782	0.99	Microtubule-associated protein 1B (MAP1B)
−1.13	S	1503;1529;1405;1431;238;265	0.54	Centrosomal protein of 170 kDa (Cep170)
−1.10	S	24;109	0.86	Transcriptional coactivator YAP1
−1.10	S	11;12;23;30	1.00	Septin-9 (Sept9)
−1.10	S	1688	1.00	Telomere-associated protein RIF1
−1.08	S	283	1.00	A-kinase anchor protein 8-like (AKAP8L)
−1.08	S	955;978;1009	0.51	Tight junction protein ZO-2 (TJP2)
−1.08	S	956;979;1010	0.51	Tight junction protein ZO-2 (TJP2)
−1.08	S	963;986;1017	0.98	Tight junction protein ZO-2 (TJP2)
−1.06	S	126;171	1.00	Polymerase I and transcript release factor (PTRF)
−1.05	S	562;568	0.75	Serine/threonine-protein kinase N1 (PKN1)

The table provides a list of hypophosphorylated proteins in CPE-overexpressing LNT229 versus corresponding Neo-control cells detected by SILAC followed by phosphoproteomic analysis. The phospho-proteins discussed in the manuscript are highlighted in **bold**. Abbreviations: H/L - log2 ratio of phospho-enrichment in “heavy”-labeled CPE-overexpressing LNT229 cells to “light”-labeled Neo-control LNT229 cells; A.a. - amno acid; Position - position of amino acid with detected phosphorylation; Loc.Prob. - localization probability.

### CPE secretion is necessary to promote RPS6 phosphorylation

**Figure 2:**
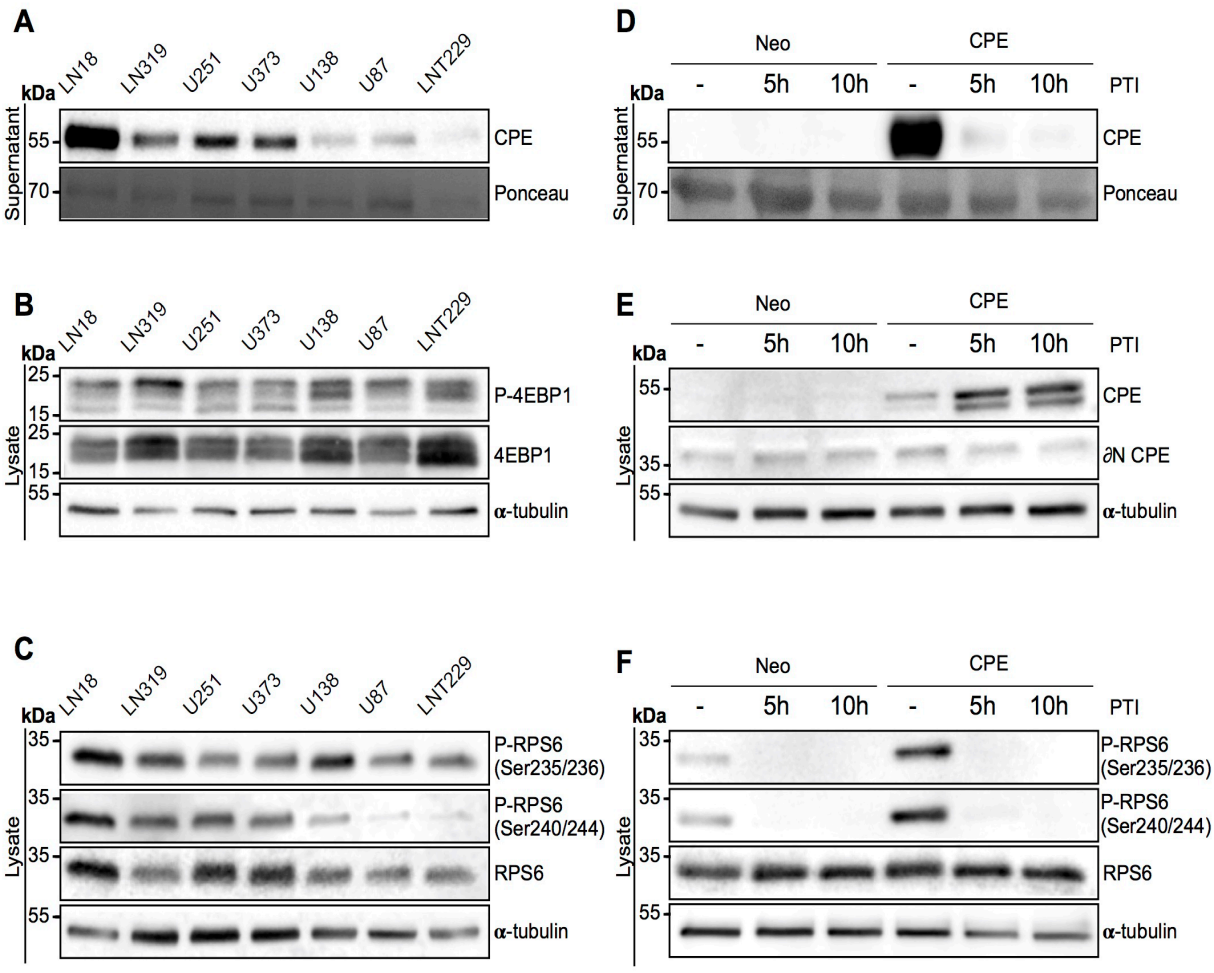
Secreted CPE induces RPS6 phosphorylation **(A-C)** Signaling profiling of 7 GBM cell lines. Immunoblot detection of **(A)** sCPE in the supernatants and **(B)** 4EBP1 and **(C)** RPS6 levels and its phosphorylation in the lysates derived from 7 GBM cell lines. Ponceau staining and α-tubulin were used as loading controls for the supernatants and lysates, respectively. The cells were serum-starved for 24h in serum-reduced medium prior to supernatant collection and lysis. Representative immunoblots are shown. **(D-F)** Signaling profiling of LNT229 Neo-control and sCPE-overexpressing cells upon inhibition of the protein transport. Detection of **(D)** sCPE in the supernatants and **(E)** CPE or **(F)** RPS6 (total and phosphorylated forms) in the lysates cells. The cells were serum-starved for 5h in serum-reduced medium (without treatment) or for 5h and 10h in serum-reduced medium with 1x protein transport inhibitor (PTI) cocktail prior to supernatant collection and lysis. Representative immunoblots are shown.

CPE exists in at least two splice-variants: full-length- (flCPE) and ∂(delta)N-CPE. flCPE is packed into secretory vesicles and is then transported into the extracellular space. To confirm that CPE indeed needs to be secreted and does not signal via intracellular pathways as well as to exclude any effect of the ∂N-splice variant of CPE, we evaluated activation of mTOR signaling in a panel of GBM cell lines, which show different levels of sCPE. In line with our former observations, no correlation between the levels of secreted CPE (Figure 2A) and phosphorylation of 4EBP1 (Figure 2B; Supplementary Figure 3A) was observed. However, there was a positive correlation between sCPE and RPS6 phosphorylation, with phosphorylation at the position Ser240/244 significantly correlating with secreted CPE (Figure 2C; Supplementary Figure 3B). Meanwhile, neither intracellular flCPE nor ∂N-CPE correlated with RPS6 and its activation (Supplementary Figure 4). We further blocked the secretory pathway with a mix of Monensin and Brefeldin A in the sCPE-overexpressing or Neo LNT229 cells and evaluated RPS6 phosphorylation. As expected, a block in secretion led to an accumulation of CPE within cells (Figure 2D, 2E). Intriguingly, phosphorylation of RPS6 but not of 4EBP1 was completely abolished when CPE secretion was inhibited (Figure 2F., Supplementary Figure 3C). This is in line with previous reports that showed 4EBP1 phosphorylation to be less sensitive to mTORC1 modulation than RPS6 phosphorylation [26]. Therefore we conclude, that CPE needs to be delivered into the extracellular space for a specific activation of mTORC1 target RPS6.

### sCPE inhibits LDHA and MCT4 expression

**Figure 3:**
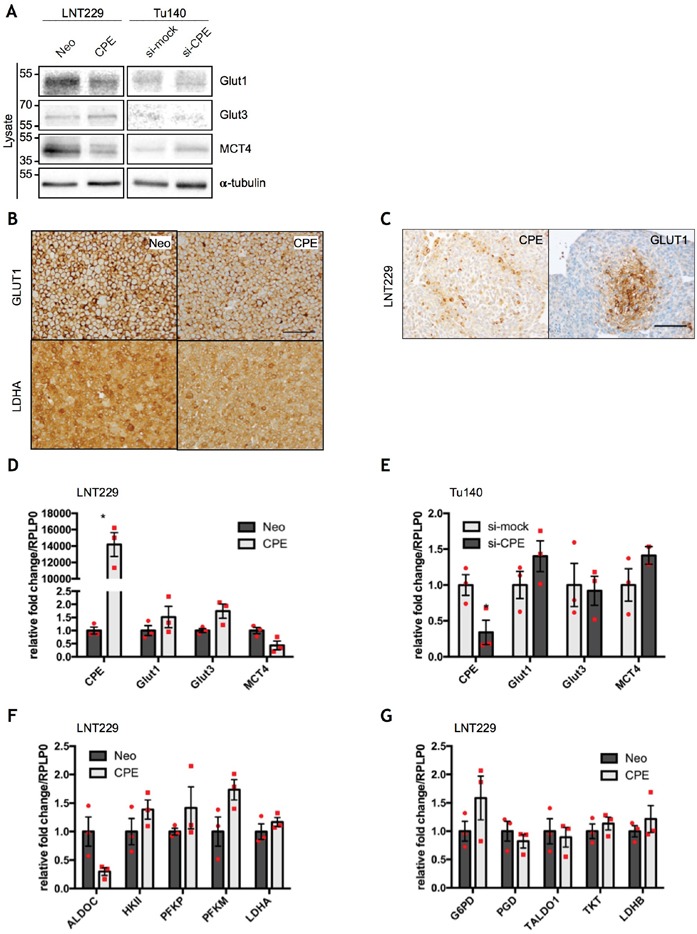
sCPE regulates glucose- and lactate transporters, but does not affect gene expression of key metabolic enzymes **(A)** Immunoblot detection of GLUT1, GLUT3 and MCT4 in the lysates of the Neo or sCPE-transfected LNT229 or Tu140 cells upon CPE knockdown. For Tu140, control siRNA (si-mock) was used as negative control. α-tubulin was used as a loading control. The cells were serum-starved for 24h in serum-reduced medium prior to lysis. A representative immunoblot is shown. **(B)** Immunohistochemical staining of GLUT1 and LDHA in the Neo or sCPE-overexpressing LNT229 cells (20x magnification, scale bar 100μm). **(C)** Immunohistochemical staining of GLUT1 in the LNT229 spheres (20x magnification, scale bar 100μm). **(D, E)** qPCR analysis of CPE, Glut1, Glut3 and MCT4 gene expression in the **(D)** Neo or sCPE-overexpressing LNT229 cells or **(E)** Tu140 cells upon CPE knockdown. Control siRNA (si-mock) was used as negative control for CPE knockdown. Red dots represent single experiments. Unpaired t-test with Welch's correction. Mean±SEM; n=3 (D: *p=0.0105; E: *p=0.0422). **(F, G)** qPCR analysis of the **(F)** glycolytic enzymes (ALDOC, HKII, PFKP, PFKM, LDHA) and **(G)** enzymes involved in the pentose-phosphate pathway (G6PD, PGD, TALDO1, TKT, LDHB) in the Neo or sCPE-overexpressing LNT229 cells. Red dots represent single experiments. Unpaired t-test with Welch's correction. Mean±SEM; n=3.

Since mTOR signaling is strongly involved in regulation of cellular metabolism and aggressive growth behavior of GBM cells requires adaptation processes to metabolic changes in the tumor microenvironment, we examined whether sCPE may also affect metabolic pathways in GBM cells. GBM cells are known to favor utilization of glucose via aerobic glycolysis, a phenomenon called “the Warburg effect” [27, 28]. We therefore first measured the intracellular levels of the main glucose- (Glut1, Glut3) and lactate transporters (MCT4) as well as levels of LDHA, which is the main enzyme involved in lactate production from pyruvate during glycolysis. Glut1, which is normally upregulated in highly glycolytic tumor cells or under hypoxia, was slightly downregulated upon sCPE overexpression in the LNT229 cell line (Figure 3A, 3B) while no gross changes were observed following siRNA-mediated knockdown of CPE in Tu140 cells (Figure 3A). Under sphere-culturing conditions, areas of CPE and Glut1 expression were mutually exclusive: Glut1 was enriched in the hypoxic center of the spheres and CPE at the outer rim, where cells have sufficient oxygen and nutrient supply (Figure 3C). Conversely, Glut3 was moderately upregulated in the sCPE-overexpressing LNT229 cells while being hardly detected in Tu140 cells independent from CPE knockdown (Figure 3A). Altogether, no broad regulation of glucose transporters was detected at protein level, most probably pointing to a cell line-specific effect. However, protein levels of the lactate transporter MCT4 were strongly reduced upon sCPE overexpression in LNT229 while being upregulated when CPE was knocked down in Tu140 (Figure 3A). Levels of the lactate-producing enzyme LDHA were diminished as well, when sCPE was overexpressed (Figure 3B). Additionally, the examined transporters were not regulated at the mRNA expression levels in both LNT229 (Figure 3D) and Tu140 (Figure 3E) cells and no considerable transcriptional regulation of the enzymes involved in glycolysis and pentose-phosphate pathway was observed as well (Figure 3F, 3G).

### sCPE enhances glucose metabolism towards TCA

**Figure 4:**
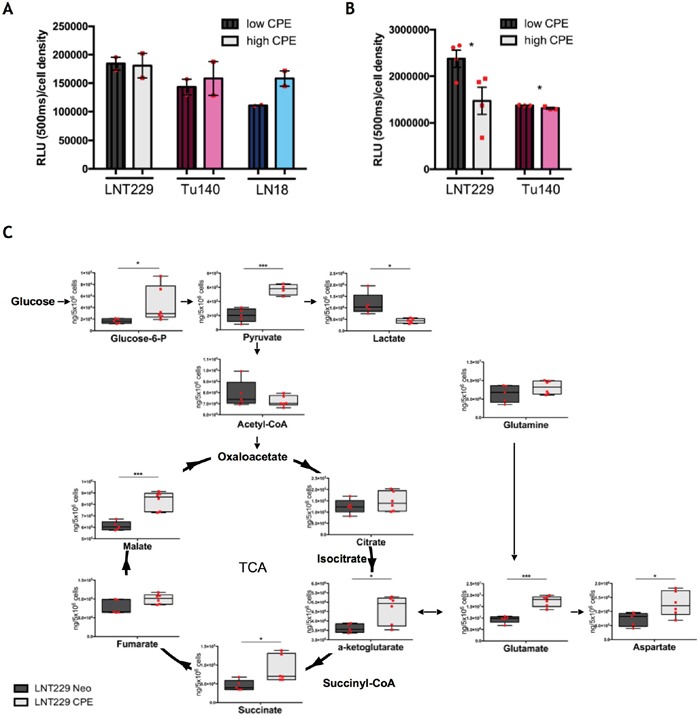
sCPE promotes a metabolic switch from Warburg effect towards TCA **(A)** Detection of 2DG uptake at 30 min time point. Dark patterned bars represent CPE-low samples (Neo for LNT229 and si-CPE for Tu140 and LN18 cells), light unpatterned bars represent CPE-high samples (sCPE-overexpression for LNT229 and control siRNA (si-mock) for LN18 and Tu140 cells). The cells were serum- and glucose-starved for 2h prior to 2DG treatment. Red dots represent single experiments. Mean±SEM; n=2. RLU - relative light unit. **(B)** Detection of extracellular lactate at 24h time point. Dark patterned bars represent CPE-low samples (Neo for LNT229 and si-CPE and Tu140 cells), light unpatterned bars represent CPE-high samples (sCPE-overexpression for LNT229 and control siRNA (si-mock) for Tu140 cells). The cells were serum- and glucose-starved for 2h prior to treatment with serum-reduced medium, containing 5mM glucose and no pyruvate. Red dots represent single experiments. Mean±SEM; n=3; LNT229 *p=0.039; Tu140 *p=0.038. RLU - relative light unit. **(C)** Quantitative analysis of TCA metabolites in the Neo or sCPE-overexpressing LNT229 cells. The cells were serum- and glucose-starved for 2h prior to treatment with serum-reduced medium, containing 5mM glucose and no pyruvate over 24h. Red dots represent single experiments. Box plots with means; N=4 for LNT229 CPE pyruvate; n=5 for LNT229 Neo glucose-6-phosphate, pyruvate, lactate, acetyl-CoA, citrate, α-ketoglutarate, succinate, fumarate, malate, glutamine, glutamate and aspartate; n=6 for LNT229 sCPE glucose-6-phosphate, lactate, acetyl-CoA, citrate, α-ketoglutarate, succinate, fumarate, malate, glutamine, glutamate and aspartate. Unpaired t-test with Welch's correction: pyruvate p=0.0004 (205207 ± 42116 ng/5 Mio cells for Neo and 570685 ± 38431 ng/5 Mio cells for sCPE, mean ± SEM); lactate p=0.0235 (1168067 ± 208831 ng/5 Mio cells for Neo and 436642 ± 40092 ng/5 Mio cells for sCPE, mean ± SEM); α-ketoglutarate p=0.0213 (3608774 ± 104542 ng/5 Mio cells for Neo and 4614385 ± 309601 ng/5 Mio cells for sCPE, mean ± SEM); malate p=0.0004 (611426 ± 17686 ng/5 Mio cells for Neo and 833821 ± 32839 ng/5 Mio cells for sCPE, mean ± SEM); glutamate p=0.0001 (9427040 ± 699446 ng/5 Mio cells for Neo and 17385165 ± 975329 ng/5 Mio cells for sCPE, mean ± SEM); aspartate p=0.0334 (726077 ± 109681 ng/5 Mio cells for Neo and 1269588 ± 180832 ng/5 Mio cells for sCPE, mean ± SEM). Non-parametric Mann-Whitney test: succinate p=0.0173 (45429 ± 6305 ng/5 Mio cells for Neo and 88300 ± 14579 ng/5 Mio cells for sCPE, mean ± SEM); glucose-6-phosphate p=0.0173 (16726 ± 1847 ng/5 Mio cells for Neo and 44848 ± 12517 ng/5 Mio cells for sCPE, mean ± SEM).

Since sCPE negatively regulates lactate-related transporters and enzymes, we proposed that sCPE might contribute to certain metabolic rearrangements in tumor cells. To assess this, we first measured glucose uptake (Figure 4A) and extracellular lactate secretion (Figure 4B) in the sCPE-overexpressing LNT229 as well as CPE-knockdown LN18 and Tu140 cells. Of note, similarly to Tu140, LN18 cells, taken for knockdown experiments, also do secrete some amounts of CPE (Supplementary Figure 1A). Whereas glucose uptake only slightly varied between the conditions, lactate secretion was heterogeneously reduced dependent on CPE in different cell lines, with only a very mild decrease in Tu140 cells (Figure 4B). To investigate the underlying mechanisms in more detail, we performed mass-spectrometry-based analysis of metabolites of glycolysis and the tricarboxylic acid cycle in the LNT229 sCPE-overexpressing versus Neo cells. Intriguingly, we observed that the pyruvate levels were unequivocally increased in sCPE-overexpressing, when compared to corresponding Neo LNT229 cells (Figure 4C). Since no external pyruvate was present in the medium, this pyruvate could only have been derived from an increased general glycolytic flux. Moreover, the intracellular lactate levels were markedly reduced (Figure 4C), corresponding to our previous observations regarding the levels of lactate-related transporters and enzymes (MCT4 and LDHA) as well as extracellular lactate levels. In addition, several key metabolites within the TCA, such as α-ketoglutarate, succinate and malate were also increased (Figure 4C), suggesting enhanced flux of glucose derivatives through the TCA cycle. Therefore, we conclude that sCPE promoted a metabolic switch from aerobic glycolysis towards the TCA cycle.

### The interplay of sCPE, mTOR inhibition and Rac1 activation impacts glioma cell migration via RPS6-Rac1 axis

**Figure 5:**
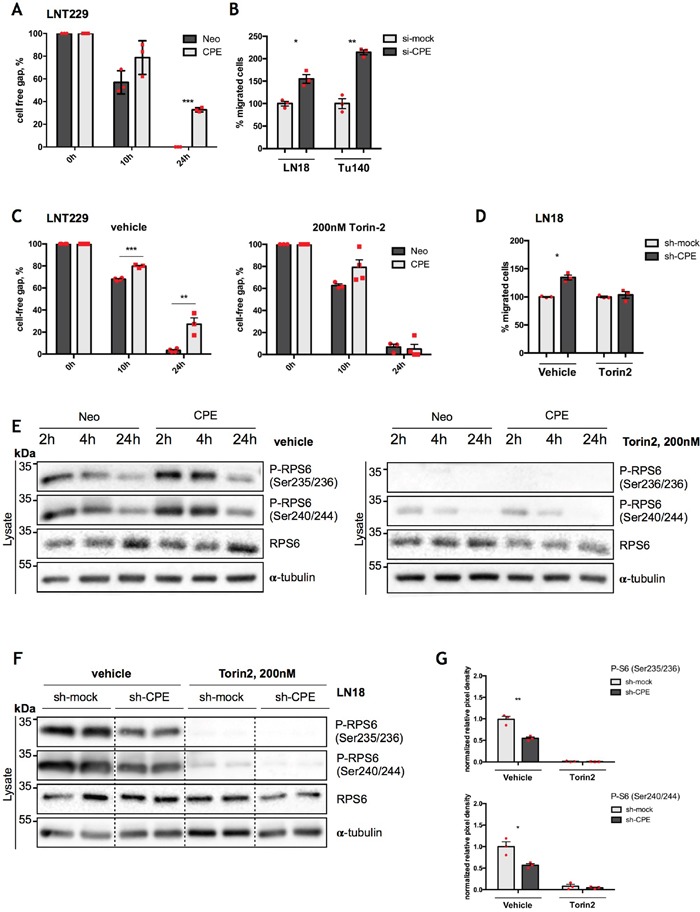
RPS6 mediates anti-migratory effects of sCPE **(A)** Assessment of cell migration by wound-healing assay in the Neo or sCPE-overexpressing LNT229 cells over 24h. Red dots represent single experiments. Multiple t-tests with Holm-Sidak correction. Mean±SEM, n=3 (***p=8.075222e-06). **(B)** Assessment of cell migration by transwell migration assay over 24h for LN18 and Tu140 cells upon transient CPE knockdown. Control siRNA (si-mock) was used as negative control. Transwell migration was analyzed 24h post RNA-interferention. Red dots represent single experiments. Unpaired t-test with Welch's correction. Mean±SEM (B: n=3, *p=0.0147; C: N=3, **p=0.0027). **(C)** Assessment of cell migration by wound-healing assay; gap closure by the Neo or sCPE-overexpressing LNT229 cells over 24h upon treatment with 200 nM Torin2. Multiple t-tests with Holm-Sidak correction. Mean±SEM (n=3, ***p=5.158002e-005, **p=0.00474945). **(D)** Assessment of cell migration by transwell migration assay over 24h for LN18 cells upon stable CPE knockdown with and without treatment with mTOR inhibitor (Torin2). Control shRNA (sh-mock) was used as negative control. Red dots represent single experiments. Unpaired t-test with Welch's correction. Mean±SEM (n=3, *p=0.0128). **(E)** Immunoblot for total and phosphorylated form of RPS6 with and without treatment with Torin2 in the lysates of the Neo or sCPE-overexpressing LNT229 cells. α-tubulin was used as a loading control. For the control (left) the cells were serum-starved for 2-, 4- or 24h in serum-reduced medium and for the mTOR inhibition (right) 200 nM Torin2 in serum-reduced medium was applied for 2-, 4- or 24h prior to lysis. A representative immunoblot is shown. **(F)** Immunoblot detection of total and phosphorylated form of RPS6 with and without treatment with mTOR inhibitor (Torin2) for 24h in the lysates of the LN18 cells upon stable CPE knockdown. Control shRNA (sh-mock) was used as negative control. α-tubulin was used as a loading control. A representative immunoblot is shown. **(G)** Quantification of densitometric measurements of immunoblotting results of phosphorylated amount of RPS6 in the LN18 cell line upon stable CPE knockdown. Red dots represent single experiments. Unpaired t-test with Welch's correction. Mean±SEM; n=3 (*p=0.0212; **p=0.0042).

**Figure 6:**
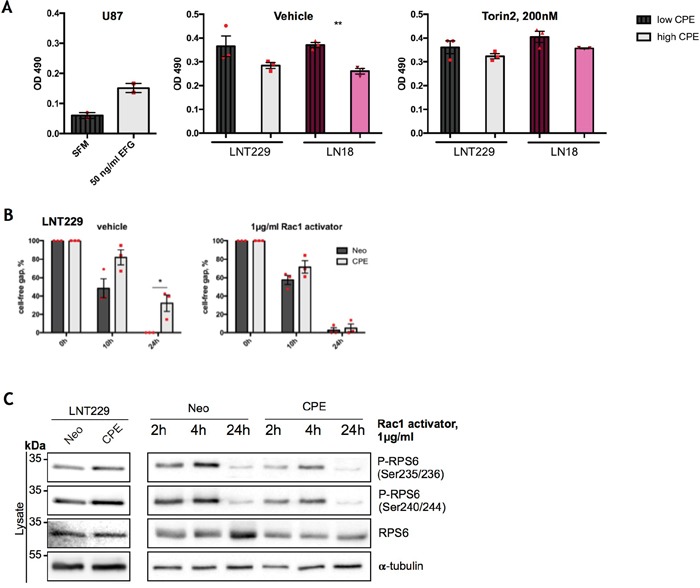
RPS6 mediates anti-migratory effects of sCPE over Rac1 **(A)** Assessment of GTP-bound Rac1 in the Neo or sCPE-overexpressing LNT229 and LN18 cells upon stable CPE knockdown, with and without treatment with Torin2. Unstimulated U87 cells treated with serum-free medium were used as a negative control while 50 ng/ml EGF stimulation of U87 - as a positive control for a Rac1-GTP. For CPE-knockdown, shRNA was used as a negative control. Red dots represent single experiments. Mean±SEM; n=3 (**p=0024). **(B)** Assessment of cell migration by wound-healing assay; gap closure by the Neo or sCPE-overexpressing LNT229 cells over 24h upon treatment with 1μg/ml Rac1-activator. Multiple t-tests with Holm-Sidak correction. Mean±SEM (n=3, *p=0.0224186). **(C)** Immunoblot detection of total and phosphorylated form of RPS6 with and without Rac1-activator in the lysates of the Neo or sCPE-overexpressing LNT229 cells. α-tubulin was used as a loading control. For the positive control (left) the cells were serum-starved for 4h in serum-reduced medium and for the Rac1 activation (right) 1μg/ml Rac1-activator in serum-reduced medium was applied for 2-, 4- or 24h prior to lysis. A representative immunoblot is shown.

Emerging evidence suggests that pathways that regulate tumor cell metabolism and migration are interconnected. Since sCPE-overexpressing GBM cells show a less migratory phenotype, as previously reported [12] (Figure 5A; Supplementary Figure 5A) and anti-migratory effects of CPE were as well consistent in the Tu140 and LN18 cells when CPE was transiently knocked down (Figure 5B), we investigated whether these effects are linked to mTOR activation. We therefore blocked mTOR with Torin2 and explored migration potential as well as RPS6 activation of either sCPE-overexpressing LNT229 cells or LN18 cells upon stable CPE knockdown. The mTOR inhibition resulted in increased migration of sCPE-overexpressing clones as well as decreased migration of LN18 cells when CPE was knocked down, thereby abolishing the anti-migratory properties of sCPE (Figure 5C, 5D; Supplementary Figure 5B). Meanwhile, reduction in RPS6 phosphorylation was detectable in both LNT229 sCPE-overexpressing and LN18 CPE-knockdown cells, and was persisting for at least 24h, covering the measured migration time (Figure 5E-5G). It has previously been shown that particularly RPS6 can be involved in the regulation of Rac-signaling in *D. melanogaster* [29]. Hence, to investigate the possible link between sCPE, RPS6 and Rac1 in glioma, we performed further functional analysis of the active (GTP-bound) form of Rac1 in the LNT229 cell line upon sCPE-overexpression as well as in LN18 cells, in which CPE was stably knocked down, with and without inhibition of mTOR. While in the overexpressing model, Rac1-GTP was only tendentially decreased in response to sCPE-overexpression, we observed a marked increase in Rac1-GTP in the LN18 cells upon CPE knockdown (Figure 6A). The differences in the active Rac1-GTP were however eliminated, when the cells were treated with Torin2. Additionally, the anti-migratory effects of sCPE in the LNT229 cells were attenuated, when the cells were theated with the Rac1 activator (Figure 6B). We further analyzed whether there was a link between the mTOR-RPS6 axis and Rac1-signaling in human glioma cells. We therefore examined the reverse Rac1-dependent regulation of RPS6. Indeed, as early as 2h after treatment with Rac1 activator, phosphorylation of RPS6 was considerably reduced in the sCPE overexpressing clones and after 24h it was barely detectable in both, Neo and sCPE clones (Figure 6C), while native RPS6 was still preserved. Altogether, we were able to detect a cross-talk between sCPE, RPS6, Rac1 and glioma cell migration.

### CPE is heterogeneously expressed *in vivo* in GBM, and correlates with RPS6

**Figure 7:**
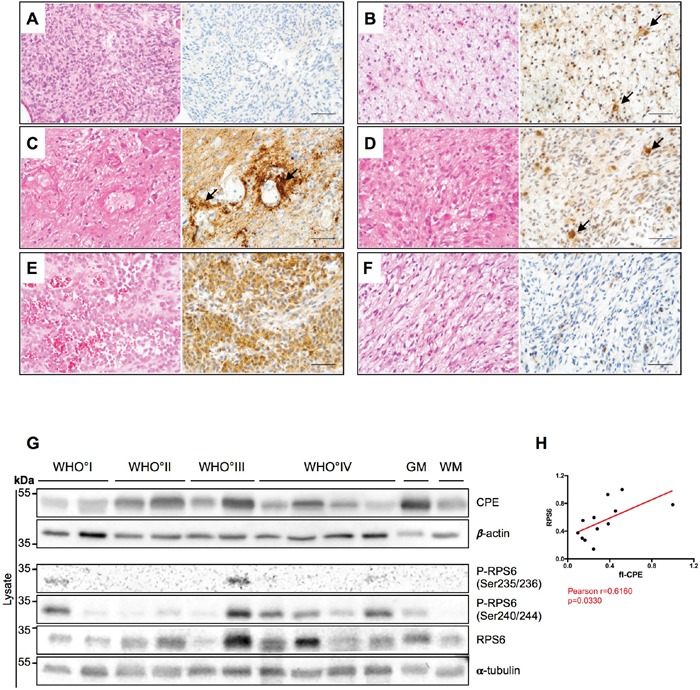
CPE is heterogeneously expressed in human gliomas **(A-F)** HE stainings (left) and CPE immunohistochemistry (right) of human glial tumors. **(A)** Human IDH wild-type glioblastoma displaying absent to very weak CPE expression. **(B)** In IDH1-mutant (IDH1_R132H) glioblastoma samples, a weak to partially moderate CPE expression was observed while on very few cells (arrows) morphologically resembling reactive astrocytes showed slightly stronger CPE expression. **(C)** In recurrent GBM, CPE was strongly accumulating in a perivascular distribution (arrows) in areas with prominent astrogliotic changes. Presumably, the respective CPE expression is at least partly localized to reactive perivascular astrocytes. **(D)** In vital tumor areas of recurrent GBM, CPE was also most strongly expressed by cells with reactive astrocytic morphology. **(E-F)** Of note, a strong heterogeneity in CEP expression was also observed within distinct tumor specimens. While areas with an **(E)** epitheloid differentiation displayed strong CPE expression in gliosarcoma, its counterparts with **(F)** sarcomatous morphology remained largely CPE-negative. (A-F: scale bars = 100μm). **(G)** A representative immunoblot for detection of CPE and RPS6 in lysates of WHO°I to WHO°IV gliomas as well as normal appearing grey (GM) and white matter (WM). α-tubulin and β-actin were used as loading controls. **(H)** Correlation analysis of densitometric measurements of immunoblotting results in the WHO setting between CPE and RPS6. Pearson coefficient and exact p-value is shown (*p<0.05).

To address the question of mutual regulation of CPE and RPS6 *in vivo*, we examined CPE expression in patient GBM samples. Of note, one can not differ between sCPE and intracellular flCPE, when examining the patient-derived specimens by immunohistochemistry or immunoblotting. In the primary IDH1/2 wild-type (IDH-wt) GBM, tumor cells did not express CPE, while IDH1/2-mutated (IDH-mut) GBM showed slight diffuse CPE staining, probably mostly deriving from reactive astrocytes (Figure 7A and 7B, respectively). The specimens from recurrent GBM showed a perivascular accumulation of CPE (Figure 7C) as well as CPE-positive cells with morphological features of reactive astrocytes (Figure 7D). Interestingly, in gliosarcoma samples, a distinct epithelial-like cell fraction was strongly positive for CPE (Figure 7E) while, in contrast, the sarcomatoid, spindle-cell like tumor cells were CPE-negative (Figure 7F). The epithelial-like tumor cells are supposed to grow in a more cohesive and therefore potentially less migratory manner. Additionally, CPE expression in human patient samples was heterogenous across the different WHO grades of gliomas (from low grade WHO°I to high grade WHO°IV), without any distinct pattern or correlation with the WHO grade (Figure 7G). We further examined, if we could also detect any correlation between CPE and RPS6 *in vivo*, and indeed, levels of CPE correlated with the levels of total RPS6 (Figure 7G, 7H). We could not detect a correlation between the CPE and active (phosphorylated) RPS6, which was probably related to the artefacts of fixed or frozen tissue [30].

## DISCUSSION

While the effects of sCPE on tumor cell migration have been reported for several entities including glioblastoma [12, 21], the exact mechanism of how this secreted protein could affect biological characteristics of tumor cells remained elusive. Recent studies pointed out that Erk1/2 and GSK3b pathways are regulated by sCPE, mainly affecting cancer cell survival [21]. Murthy *et al*., also speculated, that the inactivating phosphorylation of GSK3b could at least partly contribute a decrease in migration of fibrosarcoma cells. However studies that further investigate a potential association of anti-migratory effects of sCPE with any of those pathways are currently lacking.

Here we provide evidence for the molecular mechanisms by which sCPE reduces aerobic glycolysis and migration in GBM cells. By our unbiased phosphoproteomics approach we identified sCPE as a novel regulator of RPS6 within the mTORC1 signaling pathway. sCPE mediates the increase of the active (phosphorylated) form of RPS6 which, in turn, has two consequences: (i) enhanced mTOR effects on the cellular metabolism leading to an enhanced TCA turnover with reduced lactate levels and (ii) decreased Rac1-signaling resulting in reduced cell migration. The effects of sCPE on RPS6 phosphorylation were consistent both, in genetically-modified *in vitro* models (Figure 1B-1G) and in a panel of GBM cells (Figure 2C and Supplementary Figure 3B). Interestingly, within RPS6 especially phosphorylation at the residues Ser240/244, but not at Ser235/236 was consistently affected when CPE expression levels were altered. Ser240/244 residues are known to be a highly specific S6K kinase and therefore mTORC1 target, while Ser235/236 phosphorylation can also result from MAPK/Erk pathway activation [31, 32].

While the mass-spectrometry analysis also revealed the regulation of mTORC2 target NDRG1, it was rather inconsistent and contradictory during further validation experiments, highly depending on the cell line (Figure 1H, 1I; Supplementary Figure 2). Moreover, rather regulation of total amount of NDRG1 was observed, suggesting that also transcriptional regulation of this protein may occur. While it may appear surprising that mTORC2 complex is regulated in an rather opposite way compared to mTORC1, it has already been shown, that those two complexes might be regulated independently, since they cause diametrically opposite effects [33]. Moreover, we did not observed a relevant regulation of Akt phosphorylation at Ser473 residue (a downstream target of mTORC2 complex) in any of the models used (Supplementary Figure 6A-6D). We therefore conclude that mTORC2 is rather not involved in the sCPE effects.

The absence of obvious effects of sCPE on Akt phosphorylation at Ser473 has also consequences with regard to probable mediators of sCPE effects. Ser473 phosphorylation is known to enhance phosphorylation of the Thr308 residue [34], that is required for a complete Akt activation. In turns, fully active Akt may act as an upstream regulator of mTORC1 [35]. As we did not observe significant changes in Akt phosphorylation status, we conclude, that sCPE activates mTORC1 complex via different pathways. In our models, we could detect regulation of the AMP-activated protein kinase, catalytic α subunit (AMPKα) (Supplementary Figure 6E, 6F), however the exact involvement of in sCPE-mediated effects in glioma should be addressed in future studies.

mTORC1 complex has a diverse spectrum of functions. Although two prominent downstream targets, RPS6 and 4EBP1, are involved in the regulation of cell size and protein translation, respectively [36, 37], RPS6 has also been shown to regulate glucose homeostasis [36] and the mTOR complex itself - iron flux [38] or mitochondrial oxidative metabolism directly via YY1-PGC-1α transcription system [39, 40]. Involvement of mTOR in mitochondria-related metabolism is crucial for our study, as it is well known that the Warburg effect contributes to GBM malignancy. In concordance with that, we have shown that sCPE promotes a shift from aerobic glycolysis towards mitochondrial metabolism via mTOR activation.

While it was previously speculated that metabolic alterations and a switch to aerobic glycolysis might be a secondary effect due to a number of microenvironment changes and hypoxia adaptation, more recent data suggests an active oncogenic process and hence, one of the prominent hallmarks of cancer [41, 42]. Accordingly, mitochondria in the tumor cells are not damaged and still active, however the cells actively fuel glucose through aerobic glycolysis and that is a direct response to growth factor signaling. Although mitochondrial metabolism provides a greater net ATP production in comparison to glycolysis, rapid glycolytic turnover in the tumor cells attenuate these differences and allows for a more rapid ATP production. As suggested by Gatenby and Gilles, glycolytic phenotype might reflect evolutionary selection and must confer a growth- and spreading (through infiltration or metastasis) advantage for cancer cells [43]. Thus, glycolysis has already been linked to a more migratory tumor cell phenotype *in vitro* in glioma [11, 44], malignant melanoma [45] and breast cancer [46] and also in *in vivo* GBM model [1]. Apart from that, the main by-product of aerobic glycolysis - lactate - appears to be a pro-migratory metabolite in cancer. In glioma, lactate was able to signal through the thrombospondin1-TGFβ2 axis to directly regulate tumor cell invasion [47]. In our study we observed that sCPE led to a considerable decrease in both extra- and intracellular lactate levels, together with a decrease in lactate-producing enzyme LDHA and lactate transporter MCT4. Meanwhile, when glucose uptake was only minimally increased upon sCPE overexpression, glucose derivatives were rather shuttled towards TCA, indicating increased mitochondrial metabolism and reduced Warburg effect (Figure 4), which is also in line with the observed mTOR activation. Decreased aerobic glycolysis and lactate production can at least partly contribute to reduced migration in our glioma model. This might shed more light on the translational aspects of glioma research with regard to metabolic pathways. For instance, while mTOR is regularly considered to be pro-tumorigenic, the latter studies point out that the better understanding of the exact functions of this complex in GBM pathology is necessary, since it as well shows a tumor-unfavorable function in sensitizing tumor cells towards hypoxia-induced cell death [26]. Of note, our results are restricted to the IDH1/2 wild-type GBM while the role of sCPE in GBMs carrying IDH1/2 mutations still remains unclear. We detected upregulation of several TCA metabolites, pointing out a direct association between sCPE and TCA, however extensive measurements of citrate, isocitrate, IDH enzymatic activity and alternative products of enzymatic reaction (such as 2-hydroxyglutarate) in the CPE model are lacking in our study and should be further investigated in order to answer the question if sCPE effects are also applicable to the IDH1/2-mutated GBM. Another mechanism that contributes to the regulation of migration is connected to RPS6. Recently a novel role of RPS6 in actin dynamics regulation has been proposed. In blood-testis barrier (BTB) constitutively active quadruple phosphomimetic RPS6 led to increased actin rearrangements via Arp3 as well as downregulation and redistribution of tight junction (TJ) proteins and hence, to the BTB perturbation [48]. Although this study revealed positive regulation of actin dynamics by RPS6, the changes described by Mok et al. were (i) mediated by Akt, which could not be corroborated in our system, and (ii) could not be attributed to any of the known phosphorylation sites in RPS6. No effects specific for the Ser240/244 phosphorylation were described. However, an important feature of RPS6-Rac association has recently been described in *Drosophila melanogaster*, where degradation of RPS6 drove the activation of Rac2 GTPase and thereby promoted F-actin remodeling [29]. Hence, in *D. melanogaster* non-degraded and active RPS6 negatively regulates Rac2 GTPase. In agreement with the proposed mechanism, we showed here, that also in GBM RPS6 may be interconnected with Rac-signaling. When activated by sCPE, phosphorylated RPS6 contributed to decrease-, and when pharmacologically inhibited, dephosphorylated RPS6 resulted in an increase of GTP-bound Rac1, therefore affecting tumor cell motility. We also detected a correlation between CPE and RPS6 in the patient-derived GBM specimens across different grades of malignancy. We could not detect high levels of RPS6 phosphorylation and therefore could not measure possible connections between CPE and phosphorylated RPS6 in human tissues, most probably related to a secondary effect of long tissue fixation or air-exposure, leading to impaired phosphorylation, as we described previously [30].

**Figure 8:**
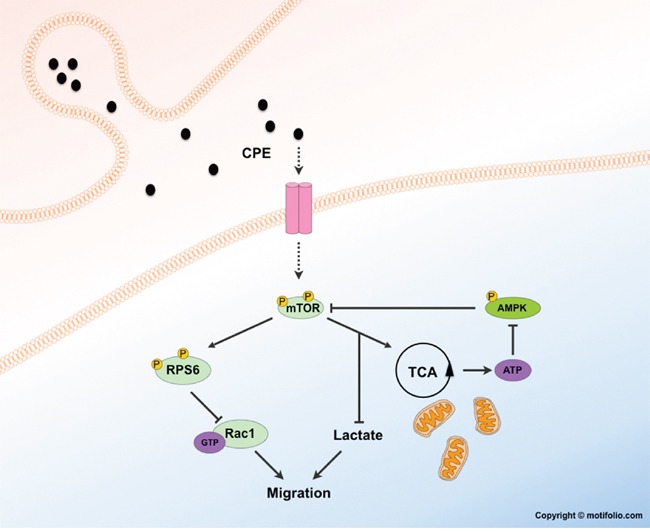
Potential mechanism of metabolism-mTOR-Rac1-migration axis regulation by sCPE

We therefore propose a model in which sCPE activates mTOR and then, both through metabolic alterations and RPS6-Rac1 axis, leads to diminished migration in GBM cells (Figure 8). As we also observed a decrease of AMPKα phosphorylation in the presence of sCPE, we suggest that hypophosphorylated AMPKα may contribute via a feedback loop system and therefore stimulate the mTOR signaling even more. However, how exactly sCPE results in activation of mTOR signaling, and specifically of RPS6, remains to be determined. One possibility is that sCPE is required for a secretion of a yet undetermined factor. That, in turn, could lead to the mentioned signaling cascades. It is well known, that CPE, for instance, is co-secreted with BDNF [49] or insulin - proteins, which serve for CPE as a cargo or as a substrate, respectively. There is evidence supporting this hypothesis and implying a possible feedback loop between RPS6 and CPE: e.g. stimulation of the neurons with BDNF led, among others, to increased RPS6 phosphorylation at the residues Ser240/244 [50]. In addition, RPS6 knock out in mice led to a considerable drop in insulin production, but not due to the decreased pancreatic beta-cell mass [36]. Alternatively, CPE itself may acts as a ligand of some yet undetermined receptor. There were trials to identify a possible receptor (among others, FGFR and Trk were studied [20]), however these studies were inconclusive so far.

Our findings shed new light onto the molecular mechanisms of regulation of GBM cell motility by sCPE and display a novel role of sCPE in tumor cell biology by functionally linking it with mTOR signaling, tumor metabolism and glioma cell migration. A deeper understanding of this interconnection might be especially relevant for designing future therapeutic strategies, to prevent therapy-induced rapid metabolic adaptation, tumor cell infiltration and hence, recurrence.

## MATERIALS AND METHODS

### Patients characteristics and tissue specimens

We investigated brain tumor samples, obtained from the University Hospital Frankfurt am Main, Germany. For western blot screening, our samples contained pilocytic astrocytomas WHO°I (n=2), diffuse astrocytomas WHO°II (n=2), anaplastic astrocytomas WHO°III (n=2), glioblastoma WHO°IV (n=4) and normal appearing grey- and white matter (n=1 for each). For immunohistochemistry, IDH1 wild-type as well as IDH1 mutated primary GBMs as well as recurrences with and without gliosarcoma components were used. The use of patients material was approved by the ethical committee of the Goethe University Frankfurt, Germany (GS04/09 and SNO-10-2014). Neuropathological diagnostics was performed by 2 experienced neuropathologists (PNH, MM) according to the current WHO classification for tumors of the central nervous system [51].

### Cell lines and reagents

Tu140 GBM low passaged (until passage 10) primary cells were established from human GBM tissue in Tübingen (Germany), LNT229, LN18 and LN319 human malignant glioma cells were provided by N. de Tribolet (Lausanne, Switzerland), other cell lines were purchased from European Collection of Authenticated Cell Cultures (ECACC, Salisbury, UK) or the American Type Culture Collection (ATCC; via LGC standards, Wesel, Germany). Mutational profiling of Tu140 GBM cells via a gene panel approach sequenced on a NextSeq 500 (Illumina, San Diego, CA, USA) revealed the following mutations: TP53:NM_001126116:exon2:c.A182C:p.H61P and RB1:NM_000321:exon11:c.1057_1058del: p.353_353del. Of note, a genetic screening revealed that U251MG and U373MG are subclasses of the same tumor [12]. All cell lines were tested for a presence of IDH1/2 mutations and all were assigned as IDH-wt. IDH1 and IDH2 sequencing was performed by standard laboratory techniques. The generation of sCPE- (from *Rattus Norvegicus*) or neomycine resistance gene alone (Neo-) expressing cells have been previously described [12]. The stably-overexpressing clones were used at passages 1-10. The transient or stable CPE knockdown Tu140 and LN18 GBM cells was generated by transfection of CPE-specific and control siPOOL siRNA (siTOOL Biotech, Martinsried, Germany) with Lipofectamine 2000 (Thermo Scientific, Waltham, MA, USA), or by lentiviral transduction of pGIPZ vectors (Dharmacon, Lafayette, USA), carrying either control or anti-CPE shRNAs, respectively. The stable CPE knockdown clones were used at passages 1-10. For detailed information about the siRNA sequences see Table 3. For culturing conditions, all cells were maintained in Dulbecco's modified Eagle's medium with 25mM Glucose (DMEM+GlutaMAX; GIBCO Life Technologies, Eggenstein, Germany) supplemented with 10% fetal calf serum (FCS; Gibco), penicillin (100 U/mL) and streptomycin (100 μg/mL) in a humidified atmosphere containing 5% CO_2_ and 21% O_2_. For spheres generation, the cells were cultured under serum-reduced conditions (DMEM+GlutaMAX supplemented with penicillin and streptomycin) for over several weeks, until 300μm spheres were formed. All reagents (if not specified otherwise) were purchased from Sigma Aldrich (Taufkirchen, Germany), protein transport inhibitor cocktail (mix of Monensin and Brefeldin A) - from eBioscience (San Diego, USA), Torin2 - from Tocris Bioscience (Bristol, UK) and Rho/Rac/cdc42 activator I - from Cytoskeleton Inc. (Denver, USA).

**Table 3:** siRNA probes (siTOOL)

siPOOL name	Sense sequence	Antisense sequence	ddG
Neg	TGTACGCGTCTCGCGATTT	AAATCGCGAGACGCGTACA	
Neg	TATACGCGGTACGATCGTT	AACGATCGTACCGCGTATA	
Neg	TTCGCGTAATAGCGATCGT	ACGATCGCTATTACGCGAA	
Neg	TCGGCGTAGTTTCGACGAT	ATCGTCGAAACTACGCCGA	
Neg	TCGCGTAAGGTTCGCGTAT	ATACGCGAACCTTACGCGA	
Neg	TCGCGATTTTAGCGCGTAT	ATACGCGCTAAAATCGCGA	
Neg	TCGCGTATATACGCTACGT	ACGTAGCGTATATACGCGA	
Neg	TTTCGCGAACGCGCGTAAT	ATTACGCGCGTTCGCGAAA	
Neg	TCGTATCGTATCGTACCGT	ACGGTACGATACGATACGA	
Neg	TTATCGCGCGTTATCGCGT	ACGCGATAACGCGCGATAA	
Neg	TCTCGTAGGTACGCGATCT	AGATCGCGTACCTACGAGA	
Neg	TCGTACTCGATAGCGCAAT	ATTGCGCTATCGAGTACGA	
Neg	TTTGCGATACCGTAACGCT	AGCGTTACGGTATCGCAAA	
Neg	TGCGTAAGGCATGTCGTAT	ATACGACATGCCTTACGCA	
Neg	TTATCGGCAGTTCGCCGTT	AACGGCGAACTGCCGATAA	
Neg	TAGCGCGACATCTATCGCT	AGCGATAGATGTCGCGCTA	
Neg	TCGTCGTATCAGCGCGTTT	AAACGCGCTGATACGACGA	
Neg	TACGCGAAACTGCGTTCGT	ACGAACGCAGTTTCGCGTA	
Neg	TCGACGATAGCTATCGCGT	ACGCGATAGCTATCGTCGA	
Neg	TCGCGTAATACGCGATCGT	ACGATCGCGTATTACGCGA	
Neg	TCGCGATAATGTTACGCGT	ACGCGTAACATTATCGCGA	
Neg	TTAACGCGCTACGCGTATT	AATACGCGTAGCGCGTTAA	
Neg	TCGCGTATAGGTAACGCGT	ACGCGTTACCTATACGCGA	
Neg	TTACGCGATCACGTAACGT	ACGTTACGTGATCGCGTAA	
Neg	TTATCGCGCGTCGCGTAAT	ATTACGCGACGCGCGATAA	
Neg	TTACGTACTAGTGCGTACT	AGTACGCACTAGTACGTAA	
Neg	TATACGCCGGTTGCGTAGT	ACTACGCAACCGGCGTATA	
Neg	TTCGCGTGCATAGCGTAAT	ATTACGCTATGCACGCGAA	
Neg	TACGCGACCTAATCGCGAT	ATCGCGATTAGGTCGCGTA	
Neg	TCGTACGCTGAACGCGTAT	ATACGCGTTCAGCGTACGA	
CPE	CCCTCATTAGCTACCTTGA	TCAAGGTAGCTAATGAGGG	3,1
CPE	GGACGAGAACTGCTCATTT	AAATGAGCAGTTCTCGTCC	4,4
CPE	CCATCTCCGTGGAAGGAAT	ATTCCTTCCACGGAGATGG	3,9
CPE	GCCTGGTGAGCCTGAATTT	AAATTCAGGCTCACCAGGC	5,9
CPE	GCTGCTTTAAATCTATCTA	TAGATAGATTTAAAGCAGC	2,1
CPE	CGGAGTTGTGAGCACTCTA	TAGAGTGCTCACAACTCCG	2,8
CPE	GCTATCTGGCAATAACAAA	TTTGTTATTGCCAGATAGC	4,2
CPE	GCCACCATGTCGCAAGAAT	ATTCTTGCGACATGGTGGC	5,7
CPE	GGCTGTCATTCATTGGATT	AATCCAATGAATGACAGCC	5,7
CPE	GTGGTAGTGCTCACGAATA	TATTCGTGAGCACTACCAC	2,9
CPE	CCTACTGGGAGGATAACAA	TTGTTATCCTCCCAGTAGG	2,4
CPE	GACTTAAATAGTTCAGTAT	ATACTGAACTATTTAAGTC	3,2
CPE	GGTTTGTGGGTCGAAGCAA	TTGCTTCGACCCACAAACC	2,5
CPE	CCCAGAATTGCATTCTGAA	TTCAGAATGCAATTCTGGG	3,8
CPE	GGGATGCAAGACTTCAATT	AATTGAAGTCTTGCATCCC	5,6
CPE	CCGCAAAGGATGGTGATTA	TAATCACCATCCTTTGCGG	4,5
CPE	GAAAGAAGGTGGTCCAAAT	ATTTGGACCACCTTCTTTC	1,8
CPE	CTGAATGAATAAAGGTTAA	TTAACCTTTATTCATTCAG	3
CPE	GTCATCGAGCTGTCCGACA	TGTCGGACAGCTCGATGAC	1,3
CPE	CCTGGAAACTATAAACTTA	TAAGTTTATAGTTTCCAGG	3,7
CPE	GTCCGTTAACACTACTTAA	TTAAGTAGTGTTAACGGAC	3,4
CPE	CCCGGGCATACTCTTCTTT	AAAGAAGAGTATGCCCGGG	5,8
CPE	CCGCCATCAGCAGGATTTA	TAAATCCTGCTGATGGCGG	4,5
CPE	CCAACGGTGGTGCTTGGTA	TACCAAGCACCACCGTTGG	1,9
CPE	GGCTTCTAGTTAGCTGCTT	AAGCAGCTAACTAGAAGCC	4,2
CPE	CCTTCAAGGTAACCCAATT	AATTGGGTTACCTTGAAGG	3,9
CPE	CGCATTCACATCATGCCTT	AAGGCATGATGTGAATGCG	3,3
CPE	GGAATAGACCACGATGTTA	TAACATCGTGGTCTATTCC	4
CPE	CCGAGGAGTTAAAGGATTT	AAATCCTTTAACTCCTCGG	4,9
CPE	GTCAACCTGATCCACAGTA	TACTGTGGATCAGGTTGAC	1,6

siRNA sequences used for RNA interference experiments.

### Measurement of cell migration

The Boyden chamber migration assay has been described in detail previously [52]. The migration was analyzed 24h after transfection over 24h for transient CPE knock down or over 48h for stable CPE knockdown by counting the migrated cells on 10 regions of interest (ROIs) per membrane at the Olympus BX1 microscope (Olympus, Tokyo, Japan) with 40x magnification objective. At least 2 membranes per biological replicate and in total 3 biological replicates were taken for statistical analysis. For the *in vitro* wound healing scratch assay, silicone ibidi inserts with a gap of 500μm were used (Ibidi, München, Germany). In brief, cells were seeded in both compartments and let attached overnight. Next day, when the cellular monolayer was formed, the inserts were removed, cells were briefly washed with PBS and full medium was applied. If migration-related signaling was manipulated, reagents were applied in the full medium to the attached cells. Photos were acquired at the 0h, 10h and 24h time points (Olympus IX70, Tokio, Japan). At least 2 technical replicates per biological replicate and in total at least 3 biological replicates were taken for statistical analysis.

### SILAC phospho-proteomics

The “EasyPhos” procedure has been previously described [53]. In brief, for stable isotope labeling by amino acids in cell culture (SILAC) LNT229-Neo control cells were maintained for at least 6 cell divisions in DMEM containing L-arginine (R0) and L-Lysine (K0) (“light” medium). The LNT229 CPE overexpressing cells were maintained similarly in DMEM containing L-Arginine-U-^13^C_6_^15^N_4_ (R10) and L-Lysine-U-^13^C_6_^15^N_2_ (K8) (“heavy” medium). DMEM was supplemented with 10% dialyzed fetal calf serum. Labeling efficiency was tested after the 5th passage by mass spectrometry analysis. Cells were harvested with trypsin, counted, mixed at 1:1 ratio (light-Neo : heavy-CPE) and lysed with guanidinium chloride (GdmCl) Buffer (6M GdmCl, 100mM Tris pH 8.5, 10mM tris(2-carboxyethil)phosphine (TCEP; Thermo Scientific, Waltham, MA, USA), 40mM 2-chloroacetamide (CAA)). Protein content was then precipitated with acetone and resuspended in TFE digestion buffer (10% 2,2,2-trifluorethanol (TFE), 100 mM ammonium bicarbonate). The samples were sonicated until homogenous suspension was formed (Bioruptor® Plus, Diagenode, Seraing, Belgium). Protein was subjected to Lys-C (Wako Pure Chemical Industries, Osako, Japan) and trypsin digestion overnight at 37°C. Phospho-peptide enrichment was carried out using TiO_2_ beads (GL Sciences, Tokyo, Japan) at 40°C for 5 min and 2000 rpm rotation followed by StageTips using styrenedivinylbenzene–reversed phase sulfonated (SDB-RPS) matrix.

### LC-MS/MS measurement and data analysis for proteomics

Peptide samples were separated on a nanoflow HPLC system (Thermo Scientific, Waltham, MA, USA) using a 90 min gradient of 5-33% acetonitrile containing 0.5% acetic acid on custom filled C18 reversed-phase columns and analyzed on a hybrid ion-trap Orbitrap mass spectrometer (Orbitrap Elite, Thermo Scientific, Waltham, MA, USA) using data-dependent acquisition selecting the most intense peaks from each full MS scan acquired in the Orbitrap for subsequent MS/MS while excluding peptides with unassigned charge states. Raw data files were processed with MaxQuant (1.5.3.8) as described previously [54, 55] using the human (UP000005640) UNIPROT database (containing 20.191 entries) and default settings including tryptic digestion allowing up to two missed cleavages, minimum peptide length of six amino acids, cysteine carbamidomethylation as fixed modification, methionine oxidation, N-terminal protein acetylation as well as serine, threonine and tyrosine phosphorylation as variable modifications, precursor mass tolerance of 20 ppm and 6 ppm for the first and main search, respectively, product ion mass tolerance of 20 ppm, revert decoy mode and standard peptide, protein and site FDR of ≤ 0.01.

### Immunoblot analysis

The cells were cultured until 90% confluence, treated respectively (complete medium-, serum-free conditions, 200 nM Torin2, 1μg/ml Rac1-activator or 1x protein transport inhibitor (mix of Monensin and Brefeldin A)) and harvested with RIPA buffer with Chaps (50 mM, Tris pH 7.5, 150 mM NaCl, 0.1% SDS, 0.5% sodium deoxycholate, 1% NP40, 0.3% Chaps). Protease and phosphatase inhibitors were added prior to lysis (Halt Protease and Phosphatase Inhibitors, Thermo Scientific, Waltham, MA, USA). The cryo-preserved patient specimens were evaluated for adequate tumor or normal brain tissue by two neuropathologists (PNH, MM) and lysed in the RIPA buffer with protease- and phosphatase inhibitors followed by homogenization as well. For generation of supernatants, the cells were cultured under serum free medium conditions. Supernatants were harvested 24h later, clarified from cell debris by centrifugation and concentrated using Amicon concentrators (Millipore, Schwalbach, Germany). Protein concentration was determined by Bradford assay (BCA, Thermo Scientific, Waltham, MA, USA). Following antibodies were used: mouse-anti-CPE (BD Bioscience, Franklin Lakes, NJ, USA), rabbit-anti-MCT4 (Novus Biologicals, Littleton, CO, USA), rabbit-anti-P-S6 ribosomal protein (S240/244) (D68F8) XP(R), rabbit-anti-P-S6 ribosomal protein (S235/236) (D57.2.2E) XP(R), mouse-anti-S6 ribosomal protein (54D2), rabbit-anti-P-4E-BP1 (T37/46) (236B4), rabbit-anti-4E-BP1 (53H11), rabbit-anti-P-AMPKα (T172) (40H9), rabbit-anti-P-Akt (S473), rabbit-anti-Akt, rabbit-anti-P-NDRG1 (T346) (D98G11) XP, rabbit-anti-NDRG1 (D8G9) XP(R) (all from Cell Signaling/New England Biolabs, Frankfurt a. M., Germany), mouse-anti-α-tubulin (Sigma Aldrich, Taufkirchen, Germany), rabbit-anti-GLUT3 (H50) (Santa Cruz, Heidelberg, Germany), rabbit-anti-β-actin and rabbit-anti-GLUT1 (Abcam, Cambridge, UK). The immunoblots were developed with the Odyssey Fc (LI-COR, Lincoln, NE, USA). For quantitative analysis of immunoblots densitometry approach was used. Pixel densities were measured by Image Studio software (LI-COR, Lincoln, NE, USA). Pixel densities of studied proteins were normalized to α-tubulin, β-actin or Ponceau for the lysates and supernatants, respectively. For the phosphorylated proteins phospho-to-total ratio was taken before normalization to the loading control. All quantitative data were modified as relative to 1 for a better data depiction.

### Total RNA isolation and quality control

Total RNAs were extracted using the peqGOLD TriFast reagent (Peqlab, Erlangen, Germany) according to the manufacturers protocol. RNA purity and integrity were monitored using NanoDrop^®^ ND-1000 spectrophotometer and Experion (BIO-RAD, Hercules, CA, USA). Only RNAs with no sign of contamination or marked degradation were considered good quality and used for further analysis.

### Quantitative RT-PCR

1μg of total RNA was reverse-transcribed using Fermentas cDNA Synthesis Kit (Thermo Scientific, Waltham, MA, USA) according to the manufacturer's protocol and real-time quantitative PCR (qPCR) was carried out using SYBR green master mix (Thermo Fisher Scientific, Waltham, MA, USA), on a MyiQ Single Color Real-Time PCR Detection System (BIO-RAD, Hercules, CA, USA). Relative mRNAs expression were quantified as ∂∂CTs: ([E∂CT(gene)/E∂CT(RPLP0)]). RPLP0 was used as a house-keeping gene. For detailed information about all primers see Table 4.

**Table 4:** quantitative RT-PCR primers

Pubmed gene ID	Gene name	Species	Direction	Sequence
NM_013128.1	CPE	Rattus Norwegicus	forward	ctcctggtcatcgagctgtct
			reverse	tcgtgtgctgtggatcaggtt
NM_001873.2	CPE	Homo Sapiens	forward	ccaccatgtcgcaagaatga
			reverse	aagctccacggtgatctcaaa
NM_001002.3	RPLP0	Homo Sapiens	forward	gagtcctggccttgtctgtgg
			reverse	tccgactcttccttggcttca
NM_006516.2	SLC2A1 (GLUT1)	Homo Sapiens	forward	gaactcttcagccagggtcca
			reverse	tccggcctttagtctcaggaa
NM_006931.2	SLC2A3 (GLUT3)	Homo Sapiens	forward	gtggaaagggcaggaagaagg
			reverse	ggccacaataaaccagggaatg
NM_003051.3	SLC16A1 (MCT1)	Homo Sapiens	forward	ccattgtggaatgctgtcctg
			reverse	atgcccatgccaatgaagaga
NM_004207.3	SLC16A3 (MCT4)	Homo Sapiens	forward	gccatgctctacgggacagg
			reverse	ggctggaagttgagtgccaaa
NM_005165.2	ALDOC	Homo Sapiens	forward	accctgggcgcttaccttct
			reverse	gctgctgctccaccatcttct
NM_000189.4	HKII	Homo Sapiens	forward	ggacttcttggccttggacct
			reverse	cgatgcactggacaatgtgg
NM_002627.4	PFKP	Homo Sapiens	forward	gaaggagtggagtgggctgct
			reverse	cgacgacctcgatgatcctgt
NM_000289.5	PFKM	Homo Sapiens	forward	gactccgagctgcctacaacc
			reverse	aaccaggcccacaatgttcag
NM_005566.3	LDHA	Homo Sapiens	forward	ggctacacatcctgggctatt
			reverse	ccttcacaaggtctgagattcc
NM_002300.6	LDHB	Homo Sapiens	forward	caagttggtatggcgtgtgct
			reverse	tcttagaattggcggtcacaga
NM_002631.2	PGD	Homo Sapiens	forward	tgccaggagggaacaaagaag
			reverse	ctcatctcccacccagtcaca
NM_006755.1	TALDO1	Homo Sapiens	forward	aagctgtcatcaacctgggaag
			reverse	cctgggcgaaggagaagagtaa
NM_001064.3	TKT	Homo Sapiens	forward	ggagctgctgaacctgaggaa
			reverse	ggtagctggccttgtcgaagt

Primers used for qRT-PCR to detect gene expression.

### Preparation of formalin-fixed paraffin-embedded (FFPE) cell pellets

The general procedure has been described in detail previously [30]. In short, the cells were cultured under standard conditions until 90% confluence. Cells were harvested with trypsin, centrifuged at 320 g in 15ml Falcon tubes to generate a cell pellet and then fixed in 4% formalin for 48h.

### Immunocytochemistry and immunohistochemistry

The general procedure has been described previously [30]. In short, immunocytochemistry was performed on freshly cut 3μm thick slides from FFPE cell pellets on the automated IHC staining system Discovery XT (Roche/Ventana, Tuscon, Arizona, USA). The following antibodies were used: rabbit-anti-CPE diluted 1:500 (Novus Biologicals); rabbit-anti-GLUT1 diluted 1:200 (Abcam), rabbit-anti-LDHA diluted 1:100 (C4B5) and rabbit-anti-P-AMPKα (T172) diluted 1:100 (40H9) (all from Cell Signaling). The staining procedure on the Discovery XT contained heat treatment of the slides (95° and 100°Celsius), CC1 cell conditioning and incubation with primary antibodies for 32 minutes. As secondary antibodies we used OMap anti-Rb HRP (Multimer HRP) for 16 minutes. As substrate we used diaminobenzidine (DAB) CM followed by a drop of H_2_O_2_. Copper was added for signal enhancement as Copper CM for 4 minutes. Slides were counterstained with hematoxylin and mounted.

### Bioluminescent assessment of glucose uptake and lactate production

Both bioluminescent assays were provided by Promega at their test-version with the manufacturer's protocols (Promega, Madison, Wisconsin, USA). The glucose uptake measurements were based on detection of 2-deoxyglucose-6-phosphate (2DG-6P) using NADPH-Glo technology: coupling oxidation of 2DG-6P with NADPH production and its subsequent bioluminescent detection using a reductase/luciferase system. The cells were seeded at the cell density 10.000 cells/well into the 96 well plate and let grown overnight. The cells were then starved in DMEM without glucose for 2h and 1mM 2DG solution was applied. The glucose uptake was measured after 30 min. After 30 min inactivation solution was added to inactivate the endogenous Glucose-6P-Dehydrogenase (G6PDH) and to prevent NADPH destruction. The measurement reagent consisted of luciferase buffer, GO buffer, NADP+, G6PDH, reductase and reductase substrate. The lactate production was similarly measured upon its oxidation coupled with NADH production and its subsequent bioluminescent detection using a reductase/luciferase system. The cells were seeded at the cell density 10.000 cells/well into the 96 well plate and let grown overnight. The cells then were starved in DMEM without glucose for 2h and DMEM with 5mM glucose and without pyruvate was applied. The extracellular lactate secretion (into medium) was measured 24h later. For that, after the incubation time, medium was collected and treated with the inactivation and neutralization solutions to inactivate the endogenous lactate dehydrogenase (LDH) and to prevent NADH degradation. The measurement reagent consisted of luciferin detection reagent, NAD, lactate dehydrogenase, reductase and reductase substrate.

### Metabolic assessment of TCA metabolites

The samples were prepared as follows: adherent cells (5 × 10^6^) were treated under serum-free conditions in DMEM medium, that contained no pyruvate and 5mM glucose for 24h, then washed twice with PBS and harvested in ice-cold 85% methanol. Samples were stored until further processing at −80°C. Prior to mass spectrometry analysis, samples were homogenized and centrifuged (10000g, 10 min, 4°C). Subsequently, supernatants were evaporated to complete dryness and resuspended in H_2_O, containing a mixture of isotop-labeled internal standards. Liquid chromatography was performed on an Agilent 1290 Infinity pump system (Agilent) using an Acquity UPLC HSS T3 column (2.1×150mm, 1.8μm, Waters). The running solvents were: A) H_2_O+0.1% formic acid; B: methanol+0.1% formic acid. Starting condition for the separation was 98% solvent A for 1.5 min, followed 3 min gradient to 100% solvent B. Mass spectrometry was performed on a QTrap 5500 mass spectrometer (Sciex, Germany) with electro spray ionization at 400°C with 4500 V in positive and −4500 V negative mode, respectively. Specific MRM transitions were monitored for every compound and normalized to appropriate isotope labeled internal standards. Data acquisition was done with Analyst 1.6.2 software (Sciex, Germany). Statistical data analysis was performed with the metaP server at the Helmholtz Zentrum München (http://metabolomics.helmholtz-muenchen.de/metap2/) [56]. Mass spectrometry measurement and bioinformatic analysis were performed by ECCPS metabolomics core facility (Frankfurt, Germany).

### Assessment of GTP-bound Rac1 protein

The analysis was done by G-LISA assay (Cytoskeleton Inc., Denver, USA) according to the manufacturers protocol. In brief, LNT229 (Neo-control or sCPE-overexpressing) or LN18 (si-mock or si-CPE) cells were cultured under standard conditions until the cells reached 40% confluence and then under serum-starving conditions for 24h. The cells were stimulates with 1% FCS in DMEM for 30 min and harvested on ice followed by snap freezing in the liquid nitrogen. For the assay test, either unstimulated (under serum-reduced conditions) or stimulated with 50 ng/ml EGF U87 GBM cell line was taken, with agreement to the protocol recommendations for Rac1 activation. Lysis buffer was used as blank and unhydrolyzed Rac1 protein was supplied with the kit as a positive control. The colorimetric signal was measured at 490 nm.

### Statistics

The figures show data obtained from at least three independent experiments as indicated in the figure legends. Each independent experiment had at least two technical replicates. Numbers and types of controls are stated for each experiment individually in the figure legends. Statistical analyses were performed using GraphPad Prism version 6.0 (GraphPad Software, CA, USA). Quantitative data was assessed for significance by unpaired student's t-test with Holm-Sidak correction for multiple comparison (for analysis of wound-healing assay, 2DG uptake and lactate production), by unpaired t-test with Welch's correction (for analysis of qPCR, TCA and transwell migration), by ratio-based paired t-test (for analysis of western blots) or by correlation analysis (for analyses across the GBM cell lines). For data, that did not pass normality test, non-parametric Mann-Whitney test was applied (alpha=0.05; *p < 0.05; **p < 0.01; ***p < 0.001).

### Datasets

*#* MassIVE = Accession Number MSV000080110.

## SUPPLEMENTARY MATERIALS FIGURES AND TABLE





## Abbreviations

2DG(−6P): 2-deoxyglucose(−6-phosphate)
4EBP1: eukaryotic translation initiation factor 4E-binding protein 1
Akt: protein kinase B
AMPK: AMP-activated protein kinase
BDNF: brain-derived neurotrophic factor
BTB: blood-testis barrier
CAA: 2-chloroacetamide
Chaps: 3-{Dimethyl[3-(4-{5,9,16-trihydroxy-2,15- dimethyltetracyclo[8.7.0.02,7.011,15] heptadecan-14-yl}pentanamido)propyl] azaniumyl}propane-1-sulfonate
CPE: carboxypeptidase E
∂N-CPE: delta N CPE (N-terminal truncated CPE)
DAB: diaminobenzidine
DMEM: dulbecco's minimal essential medium
EGF: epidermal growth factor
Erk1/2: extracellular signal-regulated kinase 1/2
FCS: fetal calf serum
FDR: false discovery rate
FFPE: formalin-fixed paraffin-embedded
FGFR: fibroblast growth factor receptor
flCPE: full length CPE
G6PDH: glucose-6-phosphate dehydrogenase
GBM: glioblastoma
GdmCl: guanidinium chloride
G-LISA: small GTPase activation assay
GLUT: glucose transporter
GSK3B: glycogen synthase kinase 3 beta
HRP: horseradish peroxidase
IDH1/2: isocitrate dehydrogenase 1/2
IHC: immunohistochemistry
LDH(A): lactate dehydrogenase (A)
MAPK: mitogen-activated protein kinase
MCT: monocarboxylate transporter
Neo: neomycine resistance
NP40: nonyl phenoxypolyethoxylethanol
mTOR: mammalian target of rapamycin
mTORC1/2: mTOR complex 1/2
NDRG1: N-Myc downstream regulated 1
PBS: phosphate buffer saline
PI3K: phosphoinositide 3-kinase
Rac1: Ras-related C3 botulinum toxin substrate 1
RIPA: radioimmunoprecipitation assay
RLU: relative light unit
RPLP0: ribosomal protein lateral stalk subunit P0
RPS6: ribosomal protein S6
sCPE: soluble (secreted) carboxypeptidase E
SDB-RPS matrix: styrenedivinylbenzene–reversed phase sulfonated matrix
SDS: sodium dodecylsulphate
SILAC: stable isotope labeling with amino acids in cell culture
TCA: tricarboxylic acid cycle
TCEP: tris(2-carboxyethil)phosphine
TFE: trifluorethanol
TGB: transforming growth factor
TJ: tight junction
Trk: tropomyosin receptor kinase
UPLC: ultra performance liquid chromatography

## References

[1] Fack F, Espedal H, Keunen O, Golebiewska A, Obad N, Harter PN, Mittelbronn M, Bähr O, Weyerbrock A, Stuhr L, Miletic H, Sakariassen PØ, Stieber D (2015). Bevacizumab treatment induces metabolic adaptation toward anaerobic metabolism in glioblastomas. Acta Neuropathol.

[2] Sanzey M, Abdul Rahim SA, Oudin A, Dirkse A, Kaoma T, Vallar L, Herold-Mende C, Bjerkvig R, Golebiewska A, Niclou SP (2015). Comprehensive analysis of glycolytic enzymes as therapeutic targets in the treatment of glioblastoma. PLoS One.

[3] Demeure K, Fack F, Duriez E, Tiemann K, Bernard A, Golebiewska A, Bougnaud S, Bjerkvig R, Domon B, Niclou SP (2016). Targeted proteomics to assess the response to anti-angiogenic treatment in human glioblastoma (GBM). Mol Cell Proteomics.

[4] Stupp R, Mason WP, van den Bent MJ, Weller M, Fisher B, Taphoorn MJ, Belanger K, Brandes AA, Marosi C, Bogdahn U, Curschmann J, Janzer RC, Ludwin SK (2005). Radiotherapy plus concomitant and adjuvant temozolomide for glioblastoma. N Engl J Med.

[5] Delgado-López PD, Corrales-García EM (2016). Survival in glioblastoma: a review on the impact of treatment modalities. Cli Transl Oncol.

[6] Giese A, Loo MA, Tran N, Haskett D, Coons SW, Berens ME (1996). Dichotomy of astrocytoma migration and proliferation. Int J Cancer.

[7] Giese A, Bjerkvig R, Berens ME, Westphal M (2003). Cost of migration: invasion of malignant gliomas and implications for treatment. J Clin Oncol.

[8] Godlewski J, Bronisz A, Nowicki MO, Chiocca EA, Lawler S (2010). MicroRNA-451: a conditional switch controlling glioma cell proliferation and migration. Cell Cycle.

[9] Dhruv HD, McDonough Winslow WS, Armstrong B, Tuncali S, Eschbacher J, Kislin K, Loftus JC, Tran NL, Berens ME (2013). Reciprocal activation of transcription factors underlies the dichotomy between proliferation and invasion of glioma cells. PLoS One.

[10] Kathagen A, Schulte A, Balcke G, Phillips HS, Martens T, Matschke J, Günther HS, Soriano R, Modrusan Z, Sandmann T, Kuhl C, Tissier A, Holz M (2013). Hypoxia and oxygenation induce a metabolic switch between pentose phosphate pathway and glycolysis in glioma stem-like cells. Acta Neuropathol.

[11] Kathagen-Buhmann A, Schulte A, Weller J, Holz M, Herold-Mende C, Glass R, Lamszus K (2016). Glycolysis and the pentose phosphate pathway are differentially associated with the dichotomous regulation of glioblastoma cell migration versus proliferation. Neuro Oncol.

[12] Höring E, Harter PN, Seznec J, Schittenhelm J, Bühring HJ, Bhattacharyya S, von Hattingen E, Zachskorn C, Mittelbronn M, Naumann U (2012). The “go or grow” potential of gliomas is linked to the neuropeptide processing enzyme Carboxypeptidase E and mediated by metabolic stress. Acta Neuropathol.

[13] Fricker LD, Snyder SH (1982). Enkephalin convertase: purification and characterisation of a specific enkephalin-synthesizing carboxypeptidase localized to adrenal chromaffin granules. Proc Natl Acad Sci USA.

[14] Fricker LD, Supattapone S, Snyder SH (1982). Enkephalin convertase: a specific enkephalin synthesizing carboxypeptidase in adrenal chromaffin granules, brain and pituitary gland. Life Sci.

[15] Fricker LD, Snyder SH (1983). Purification and characterization of enkephalin convertase, an enkephalin-synthesizing carboxypeptidase. J Biol Chem.

[16] Loh YP, Snell CR, Cool DR (1997). Receptor-mediated targeting of hormones to secretory granules: role of carboxypeptidase. E. Trends Endocrinol Metab.

[17] Park JJ, Cawley NX, Loh YP (2008). Carboxypeptidase E cytoplasmic tail-driven vesicle transport is key for activity-dependent secretion of peptide hormones. Mol Endocrinol.

[18] Lou H, Park JJ, Cawley NX, Sarcon A, Sun L, Adams T, Loh YP (2010). Carboxypeptidase E cytoplasmic tail mediates localization of synaptic vesicles to the pre-active zone in hypothalammic pre-synaptic terminals. J Neurochem.

[19] Murthy SR, Thouennon E, Li WS, Cheng Y, Bhupatkar J, Cawley NX, Lane M, Merchenthaler I, Loh YP (2013). Carboxypeptidas E protects hippocampal neurons during stress in male mice by up-regulating prosurvival BCL2 protein expression. Endocrinology.

[20] Cheng Y, Cawley NX, Loh YP (2013). Carboxypeptidase E/NF-1a1: a new neurotrophic factor against oxidative stress-induced apoptotic cell death mediated by ERK and PI3-K/Akt pathways. PLoS One.

[21] Murthy SR, Dupart E, Al-Sweel N, Chen A, Cawley NX, Loh YP (2013). Carboxypeptidase E promotes cancer cell survival, but inhibits migration and invasion. Cancer Lett.

[22] Avruch J, Belham C, Weng Q, Hara K, Yonezawa K (2001). The p70 S6 kinase integrates nutrient and growth signals to control translational capacity. Prog Mol Subcell Biol.

[23] García-Martínez JM, Alessi DR (2008). mTOR complex 2 (mTORC2) controls hydrophobic motif phosphorylation and activation of serum- and glucocorticoid-induced protein kinase 1 (SGK1). Biochem J.

[24] Arber S, Barbayannis FA, Hanser H, Schneider C, Stanyon CA, Bernard O, Caroni P (1998). Regulation of actin dynamics through phosphorylation of cofilin by LIM-kinase. Nature.

[25] Wang M, Zhu X, Sha Z, Li N, Li D, Chen L (2015). High expression of kinesin light chain-2, a novel target of miR-125b, is associated with poor clinical outcome of elderly non-small-cell lung cancer patients. Br J Cancer.

[26] Ronellenfitsch MW, Brucker DP, Burger MC, Wolking S, Tritschler F, Rieger J, Wick W, Weller M, Steinbach JP (2009). Antagonism of the mammalian target of rapamycin selectively mediates metabolic effects of epidermal growth factor receptor inhibition and protects human malignant glioma cells from hypoxia-induced cell death. Brain.

[27] Warburg O (1956). On the origin of cancer cells. Science.

[28] Vander Heiden MG, Cantley LC, Thompson CB (2009). Understanding the Warburg effect: the metabolic requirments of cell proliferation. Science.

[29] Xiao H, Wang H, Silva EA, Thompson J, Guillou A, Yates JR, Buchon N, Franc NC (2015). The Pallbearer E3 ligase promotes actin remodeling via Rac in efferocytosis by degrading the ribosomal protein S6. Dev Cell.

[30] Harter PN, Jennewein L, Baumgarten P, Ilina E, Burger MC, Thiepold AL, Tichy J, Zörnig M, Senft C, Steinbach JP, Mittelbronn M, Ronellenfitsch MW (2015). Immunohistochemical assessment of phosphorylated mTORC1-pathway proteins in human brain tumors. PLoS One.

[31] Pende M, Um SH, Mieulet V, Sticker M, Goss VL, Mestan J, Mueller M, Fumagalli S, Kozma SC, Thomas G (2004). S6K1(−/−)/S6K2(−/−) mice exhibit perinatal lethality and rapamycin-sensitive 5′-terminal oligopyrimidine mRNA translation and reveal a mitogen-activated protein kinase-dependent S6 kinase pathway. Mol Cell Bio.

[32] Biever A, Valjent E, Puighermanal E (2015). Ribosomal protein S6 phosphorylation in the nervous system: from regulation to function. Front Mol Neurosci.

[33] Miao L, Yang L, Huang H, Liang F, Ling C, Hu Y (2016). mTORC1 is necessary but mTORC2 and GSK3Δ are inhibitory for AKT3-induced axon regeneration in the central nervous system. Elife.

[34] Scheid MP, Marignani PA, Woodgett JR (2002). Multiple phosphoinositide 3-kinase-dependent steps in activation of protein kinasee B. Mol Cell Biol.

[35] Sabatini DM (2006). mTOR and cancer: insights into a complex relationship. Nature Rev Cancer.

[36] Ruvinsky I, Sharon N, Lerer T, Cohen H, Stolovich-Rain M, Nir T, Dor Y, Zisman P, Meyuhas O (2005). Ribosomal protein S6 phosphorylation is a determinant of cells size and glucose homeostasis. Genes Dev.

[37] Thoreen CC, Chantranupong L, Keys HR, Wang T, Gray NS, Sabatini DM (2012). A unifying model for mTORC1-mediated regulation of mRNA translation. Nature.

[38] Bayeva M, Khechaduri A, Puig S, Chang HC, Patial S, Blackshear PJ, Ardehali H (2012). mTOR regulates cellular iron homeostasis through Tristetraprolin. Cell Metab.

[39] Schieke SM, Phillips D, McCoy JP, Aponte AM, Shen RF, Balaban RS, Finkel T (2006). The mammalian target of rapamycin (mTOR) pathway regulates mitochondrial oxygen consumption and oxidative capacity. J Biol Chem.

[40] Cunningham JT, Rodgers JT, Arlow DH, Vazquez F, Mootha VK, Puigserver P (2007). mTOR controls mitochondrial oxidative function through a YY1-PGC-1alpha transcriptional complex. Nature.

[41] Hanahan D, Weinberg RA (2011). Hallmarks of cancer: the next generation. Cell.

[42] Ward PS, Thompson CB (2012). Metabolic reprogramming: a chancer hallmark even Warburg did not anticipate. Cancer Cell.

[43] Gatenby RA, Gillies RJ (2004). Why do cancers have high aerobic glycolysis?. Nat Rev Cancer.

[44] Ramão A, Gimenez M, Laure HJ, Izumi C, Vida RC, Oba-Shinjo S, Marie SK, Rosa JC (2012). Changes in the expression of proteins associated with aerobic glycolysis and cell migration are involved in tumorigenic ability of two glioma cell lines. Proteome Sci.

[45] Bettum IJ, Gorad SS, Barkovskaya A, Pettersen S, Moestue SA, Vasiliauskaite K, Tenstad E, Øyjord T, Risa Ø, Nygaard V, Mælandsmo GM, Prasmickaite L (2015). Metabolic reprogramming supports the invasive phenotype in malignant melanoma. Cancer Lett.

[46] Zhou L, Jiang S, Fu Q, Smith K, Tu K, Li H, Zhao Y (2016). FASN, ErbB2-mediated glycolysis is required for breast cancer cell migration. Oncol Rep.

[47] Seliger C, Leukel P, Moeckel S, Jachnik B, Lottaz C, Kreutz M, Brawanski A, Proescholdt M, Bogdahn U, Bosserhoff AK, Vollmann-Zwerenz A, Hau P (2013). Lactate-modulated induction of THBS-1 activates transforming growth factor (TGF)-beta2 and migration of glioma cells in vitro. PLoS One.

[48] Mok KW, Chen H, Lee WM, Cheng CY (2015). rpS6 regulates blood-testis barrier dynamics through Arp3-mediated actin microfilament organization in rat sertoli cells. An *in vitro* study. Endocrinology.

[49] Lou H, Kim SK, Zaitsev E, Snell CR, Lu B, Loh YP (2005). Sorting and activity-dependent secretion of BDNF require interaction of a specific motif with the sorting receptor carboxypeptidase e. Neuron.

[50] Takei N, Inamura N, Kawamura M, Namba H, Hara K, Yonezawa K, Nawa H (2004). Brain-derived neurotrophic factor induces mammalian target of rapamycin-dependent local activation of translation machinery and protein synthesis in neuronal dendrites. J Neurosci.

[51] Louis DN, Ohgaki H, Wiestler OD, Cavenee WK, Burger PC, Jouvet A, Scheithauer BW, Kleihues P (2007). The 2007 WHO classication of tumours of the central nervous system. Acta Neuropathol.

[52] Seznec J, Silkenstedt B, Naumann U (2011). Therapeutic effects of the Sp1 inhibitor mithramycin A in glioblastoma. J Neuro-Oncol.

[53] Humphrey SJ, Azimifar SB, Mann M (2015). High-throughput phosphoproteomics reveals in vivo insulin signaling dynamics. Nat Biotechnol.

[54] Cox J, Mann M (2008). MaxQuant enables high peptide identification rates, individualized p.p.b.-range mass accuracies and proteome-wide protein quantification. Nat Biotechnol.

[55] Cox J, Neuhauser N, Michalski A, Scheltema RA, Olsen JV, Mann M (2011). Andromeda: a peptide search engine integrated into the MaxQuant environment. J Proteome Res.

[56] Kastenmüller G, Römisch-Margl W, Wägele B, Altmaier E, Suhre K (2011). MetaP-server: A web-based metabolomics data analysis tool. J Biomed Biotechnol.

