# Supplementary material for: Effects of soluble CPE on glioma cell migration are associated with mTOR activation and enhanced glucose flux
Source: Oncotarget. 2017 Jun 27;8(40):67567–91. doi: 10.18632/oncotarget.18747 (PMC5620194; doi:10.18632/oncotarget.18747)
Supplement: Supplementary file 1 [file oncotarget-08-67567-s001.pdf]

Effects of soluble CPE on glioma cell migration are associated with mTOR activation and enhanced glucose flux

SUPPLEMENTARY MATERIALS

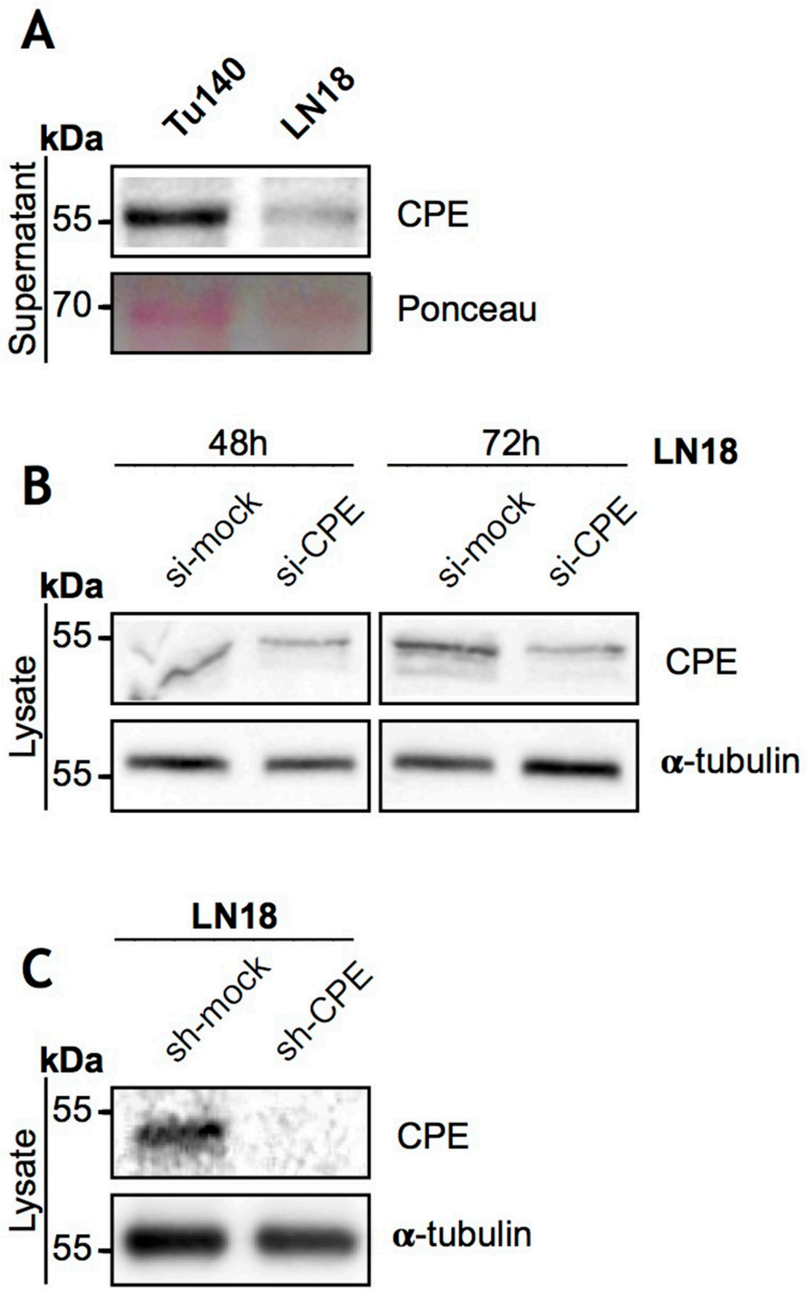

Supplementary Figure 1: Tu140 and LN18 cells secrete CPE. (A) Immunoblot detection of sCPE obtained from the supernatants of the parental Tu140 and LN18 GBM cells. Ponceau staining was used as a loading control. The cells were serum-starved for 24h in serum-reduced medium prior to supernatant collection. A representative immunoblot is shown. **(B, C)** Representative immunoblots for detection of CPE in the **(B)** transient or **(C)** stable CPE knockdown in the LN18 cells. Control si- or shRNA (si-mock/sh-mock) were used as negative controls. α-tubulin was used as a loading control.

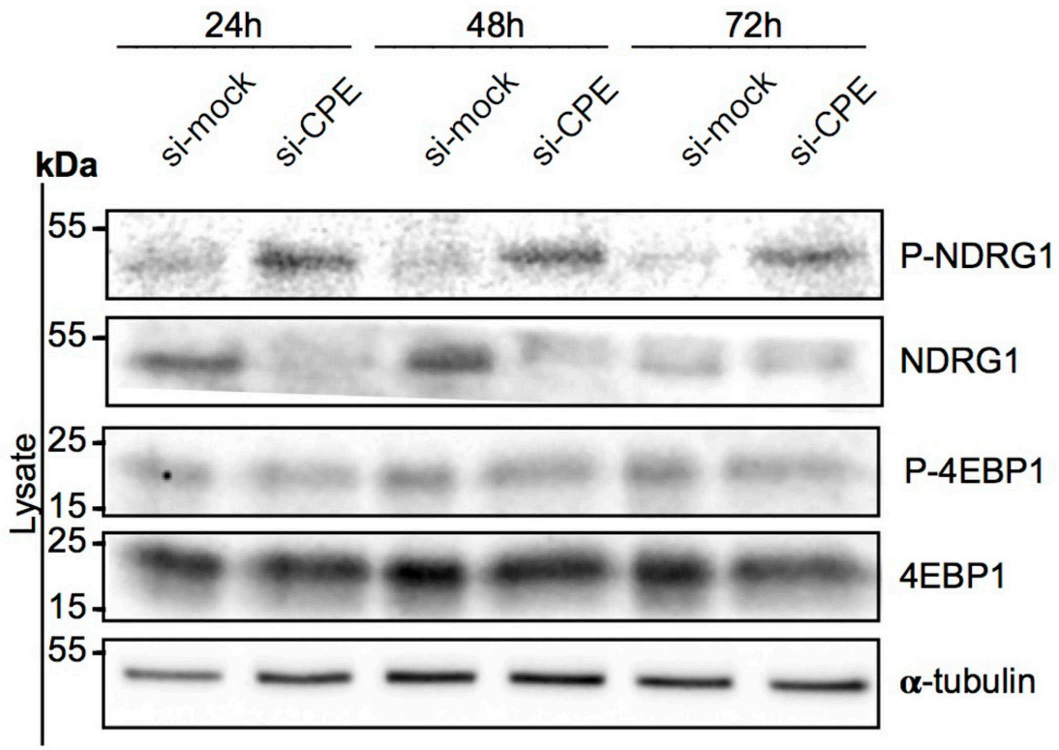

Supplementary Figure 2: Signaling profiling of Tu140 cells. Representative immunoblots for detection of phosphorylated and total amounts of NDRG1 and 4EBP1 in the CPE-knockdown primary GBM Tu140 cells. Control siRNA (si-mock) was used as negative control. α-tubulin was used as a loading control.

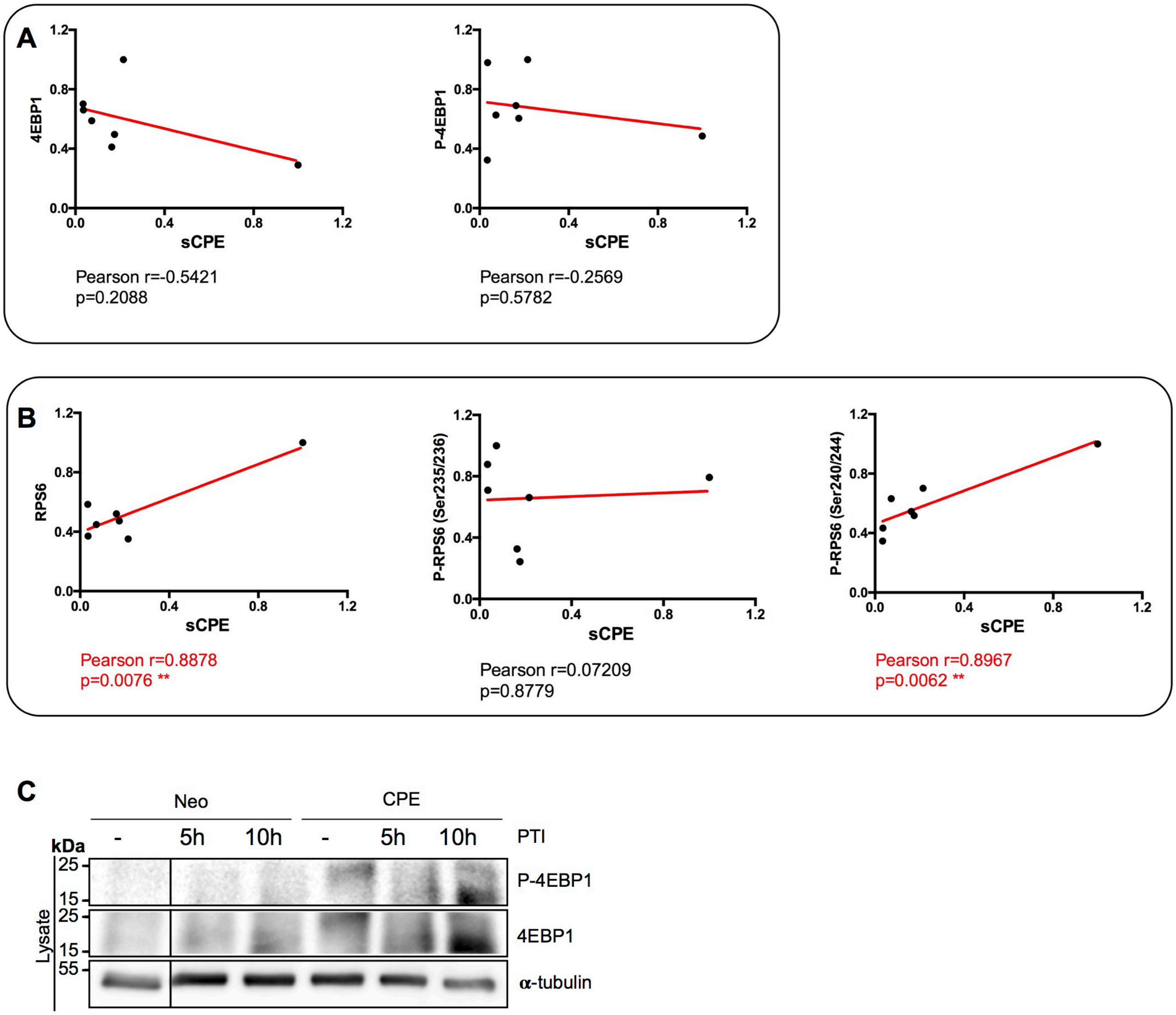

Supplementary Figure 3: CPE secretion is necessary to activate RPS6. **(A, B)** Correlation analysis of densitometric measurements of immunoblotting results in 7 GBM cell lines between sCPE and total as well as phosphorylated forms of **(A)** 4EBP1 and **(B)** RPS6. Pearson coefficients and exact p-values are shown (**p<0.01). **(C)** Detection of 4EBP1 and its phosphorylation in the lysates of the Neo or sCPE-overexpressing LNT229 cell line. The cells were serum-starved for 5h in serum-reduced medium (without treatment) or for 5h and 10h in serum-reduced medium with 1x protein transport inhibitor (PTI) cocktail prior to lysis. A representative immunoblot is shown. α-tubulin was used as a loading control.

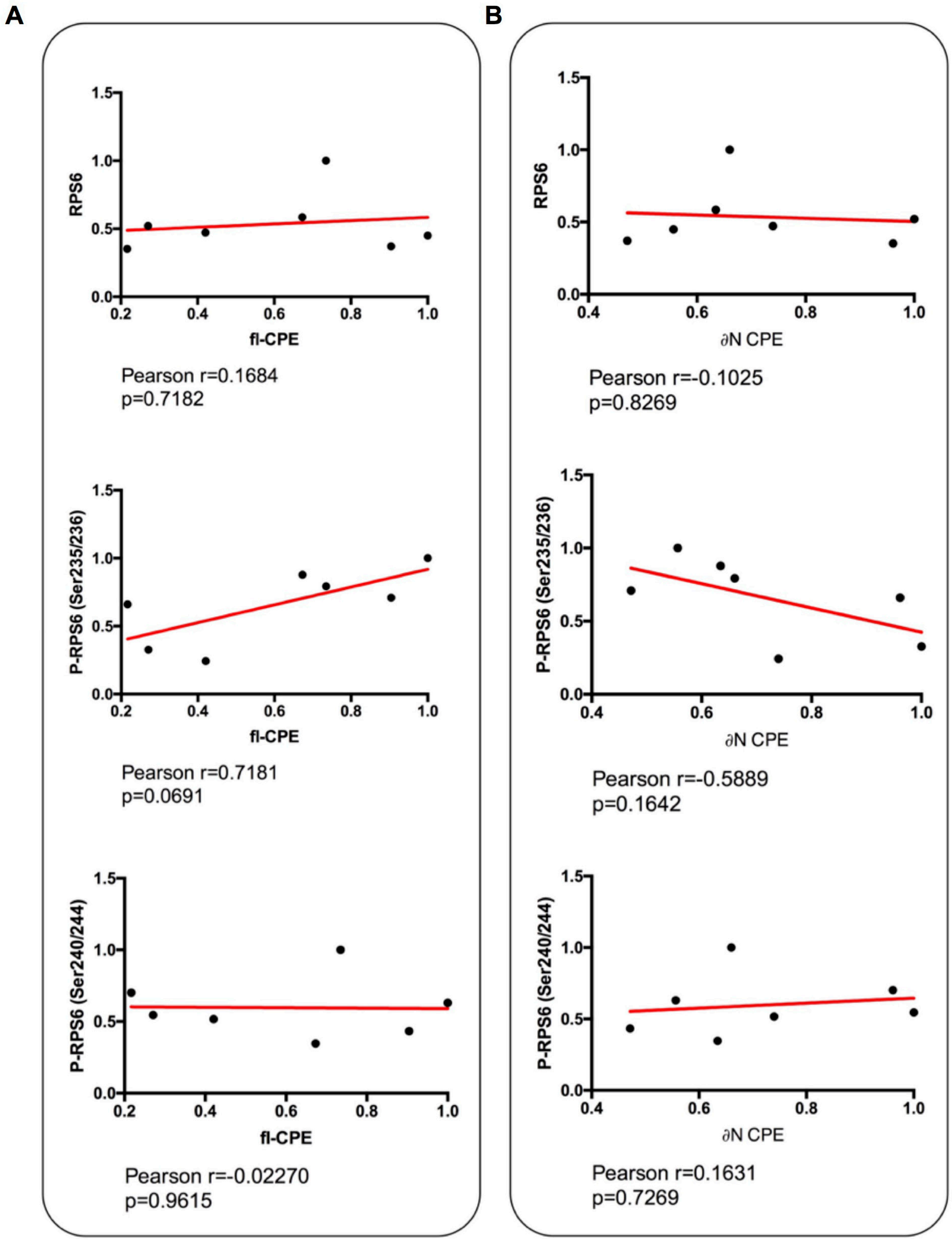

Supplementary Figure 4: flCPE and ∂N-CPE do not correlate with RPS6 in GBM cells. **(A, B)** Correlation analysis of densitometric measurements of immunoblotting results in 7 GBM cell lines between **(A)** flCPE and RPS6 and its phosphorylation form or **(B)** between ∂N-CPE and RPS6.

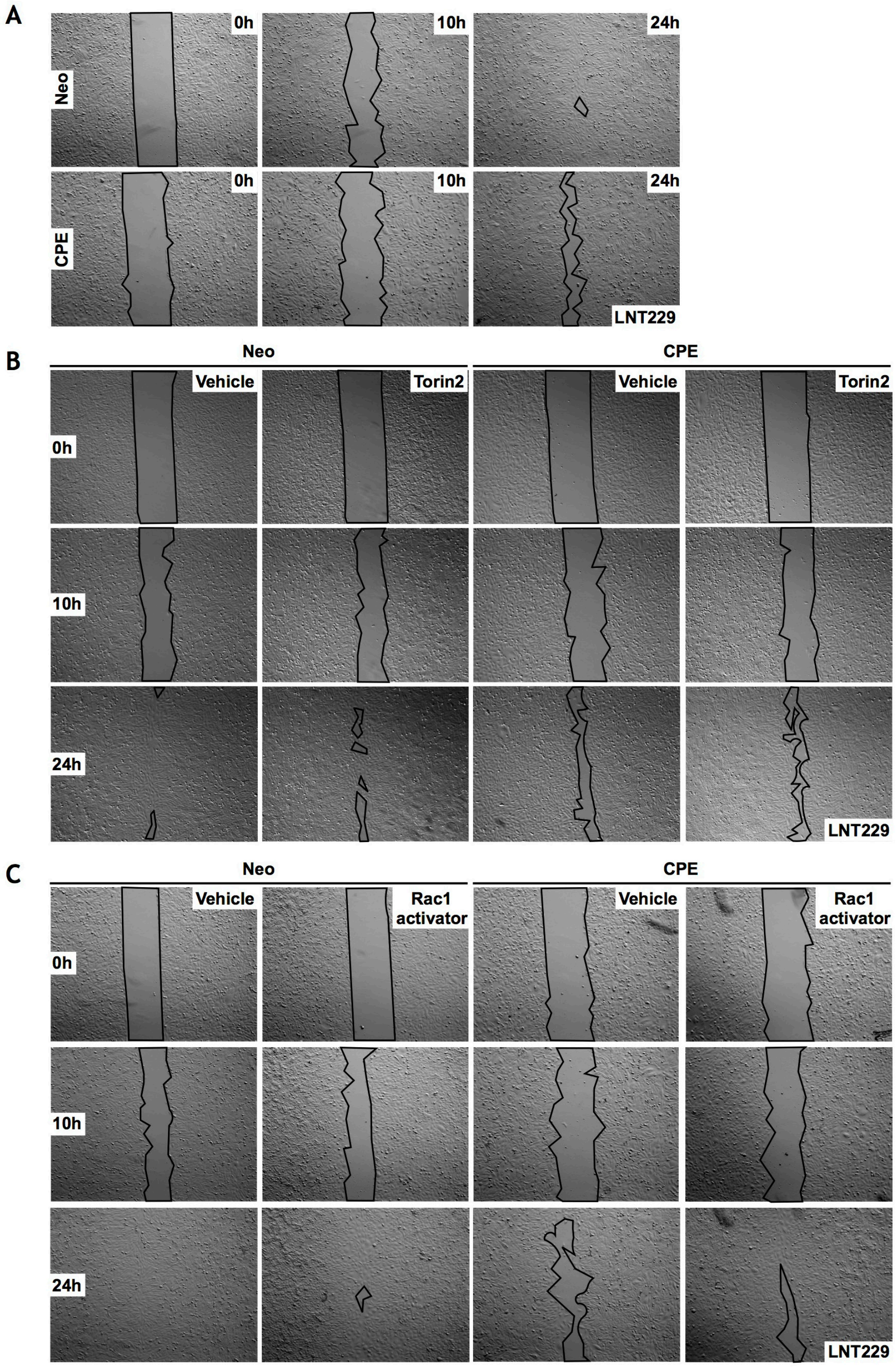

Supplementary Figure 5: Wound-healing assay in LNT229 sCPE-overexpressing cells. (A-C) Representative experiments, showing closure of the cell-free gap by sCPE-overexpressing or corresponding control (Neo) LNT229 cells over 24h under **(A)** standard conditions, **(B)** treatment with Torin2 and **(C)** treatment with Rac1 activator.

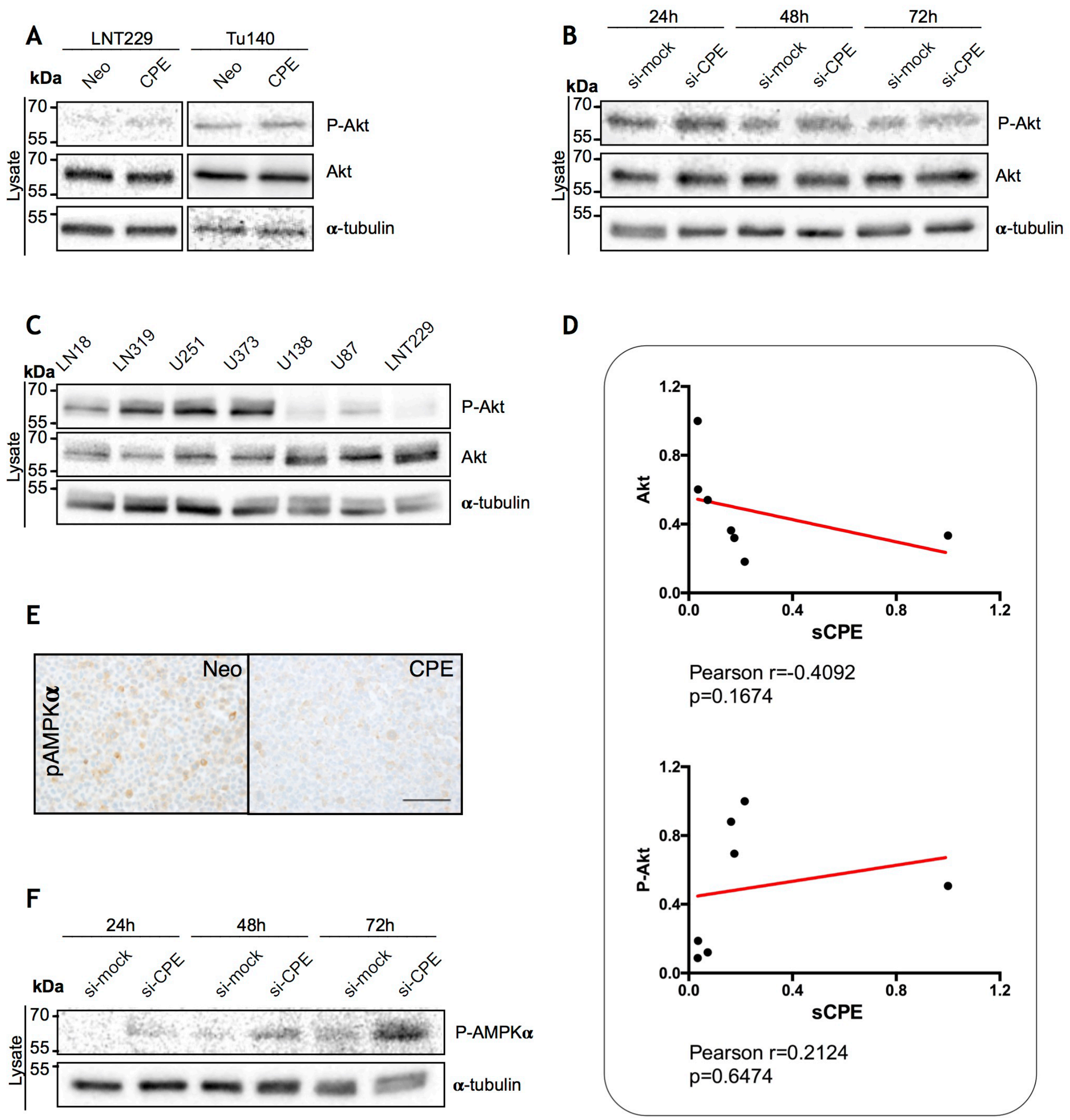

Supplementary Figure 6: Potential intracellular mediators of sCPE effects on glioma cell migration. (A) Representative immunoblots for detection of phosphorylated and total amounts of Akt in the sCPE-overexpressing vs. Neo LNT229 and Tu140 cell lysates. α-tubulin was used as a loading control. The cells were serum-starved for 24h in serum-reduced medium prior to lysis. **(B)** Representative immunoblots for detection of phosphorylated and total amounts of Akt in the CPE-knockdown primary GBM Tu140 cells. Control siRNA (si-mock) was used as negative control. α-tubulin was used as a loading control. **(C)** Signaling profiling of 7 GBM cell lines. Immunoblot detection of Akt levels and its phosphorylation in the lysates derived from 7 GBM cell lines. α-tubulin were used as loading control. The cells were serum-starved for 24h in serum-reduced medium prior to lysis. Representative immunoblots are shown. **(D)** Correlation analysis of densitometric measurements of immunoblotting results in 7 GBM cell lines between sCPE and total as well as phosphorylated forms of Akt. Pearson coefficients and exact p-values are shown. **(E)** Immunohistochemical staining of pAMPKα in the Neo or sCPE-overexpressing LNT229 cells (20x magnification, scale bar 100μm). **(F)** A representative immunoblot for detection of phosphorylated AMPKα in the CPE-knockdown primary GBM Tu140 cells. Control siRNA (si-mock) was used as negative control. α-tubulin was used as a loading control.

Supplementary Table 1: MS-based phosphoproteomic analysis in LNT229 glioma cells upon sCPE overexpression

See Supplementary File 1
